# Antimicrobial Peptide Potency is Facilitated by Greater Conformational Flexibility when Binding to Gram-negative Bacterial Inner Membranes

**DOI:** 10.1038/srep37639

**Published:** 2016-11-22

**Authors:** Sarah-Beth T. A. Amos, Louic S. Vermeer, Philip M. Ferguson, Justyna Kozlowska, Matthew Davy, Tam T. Bui, Alex F. Drake, Christian D. Lorenz, A. James Mason

**Affiliations:** 1 Institute of Pharmaceutical Science, King’s College London, Franklin-Wilkins Building, 150 Stamford Street, London, SE1 9NH, United Kingdom; 2 Department of Physics, King’s College London, London WC2R 2LS, United Kingdom

## Abstract

The interaction of antimicrobial peptides (AMPs) with the inner membrane of Gram-negative bacteria is a key determinant of their abilities to exert diverse bactericidal effects. Here we present a molecular level understanding of the initial target membrane interaction for two cationic α-helical AMPs that share structural similarities but have a ten-fold difference in antibacterial potency towards Gram-negative bacteria. The binding and insertion from solution of pleurocidin or magainin 2 to membranes representing the inner membrane of Gram-negative bacteria, comprising a mixture of 128 anionic and 384 zwitterionic lipids, is monitored over 100 ns in all atom molecular dynamics simulations. The effects of the membrane interaction on both the peptide and lipid constituents are considered and compared with new and published experimental data obtained in the steady state. While both magainin 2 and pleurocidin are capable of disrupting bacterial membranes, the greater potency of pleurocidin is linked to its ability to penetrate within the bacterial cell. We show that pleurocidin displays much greater conformational flexibility when compared with magainin 2, resists self-association at the membrane surface and penetrates further into the hydrophobic core of the lipid bilayer. Conformational flexibility is therefore revealed as a key feature required of apparently α-helical cationic AMPs for enhanced antibacterial potency.

The role of antimicrobial peptides (AMPs) is increasingly recognized as being multifaceted^1^, with host defense abilities acknowledged in addition to their much studied bactericidal activity^2^. Our understanding of this latter activity is itself being revised as a more sophisticated understanding of how such peptides operate^3^, and how bacteria respond to such challenges^4^, develops. Driven by the need to discover new antibiotics to combat the emergence of resistant organisms^5^,^6^,^7^, the focus of much research has been to discover the mechanism of action of a given peptide, in particular cationic amphipathic AMPs, and then improve certain features of the peptide to enhance its bactericidal potency. This approach has had some success but the expanding scope of AMP structures and the proliferation of numerous models for their mechanism of action^8^ hint at an underlying problem; namely that the potential targets for such peptides, and the ways of interfering with bacterial integrity and machinery, are so numerous and diverse that perfecting such “dirty drugs” becomes increasingly empirical; modification of an AMP to enhance one known beneficial property, without understanding of the wider consequences on the AMP behavior, may compromise other beneficial features and hence improvements in AMPs may be serendipitous and/or fall short of their full potential.

Molecular level information that explains bactericidal potency therefore has the potential to identify the bactericidal strategies with the greatest likelihood of success and scope for peptide improvement. According to their cationic and amphipathic nature, most AMPs are expected to interact with bacterial membranes and, whether bacterial death is ultimately caused by this interaction or interactions with intracellular machinery, the outcome of the AMP-membrane interaction is likely a key determinant of antibacterial potency. Unfortunately, molecular level information on peptide-membrane interactions is scarce with the presence of such large molecular aggregates complicating the application of many traditional experimental techniques. To address this, molecular dynamics (MD) simulations have been applied to study the interaction of AMPs with membranes e.g. refs 9, 10, 11, 12, 13, 14 while the available computer models increasingly resemble the membranes of their target organisms^15^,^16^,^17^.

Recently our attention has been drawn to two cationic amphipathic peptides, pleurocidin (from *Pleuronectes americanus*) and magainin 2 (from *Xenopus laevis*), that seemingly share a number of physico-chemical properties, adopting near identical secondary structures in traditional membrane mimetic media^17^.

Interestingly however, these peptides have a ten-fold difference in potency towards Gram-negative bacteria such as *Escherichia coli* and *Pseudomonas aeruginosa*, as determined *in vitro*, and have profoundly different effects on the target cells^4^,^17^. In a parallel, systems biology approach we investigated how *E. coli* respond at the level of the metabolome and transcriptome to challenge with sub-lethal concentrations of pleurocidin and magainin 2^4^. This study enabled comparison of gene ontology terms for genes differentially expressed in response to challenge with the two AMPs. When the terms relating to cellular component were compared, the established view that magainin 2 largely acts on the plasma membrane of Gram-negative bacteria and that this is a common target for pleurocidin was confirmed (Fig. 1). However, pleurocidin additionally impacted on a large number of intracellular biological processes indicating a multifaceted antibacterial strategy and suggesting that the greater potency of pleurocidin is due to its greater ability to penetrate the inner membrane of Gram-negative bacteria. This previous work therefore suggests that there may be subtle differences in how these two AMPs interact with the plasma membrane of Gram-negative bacteria. Indeed, previous experiments have shown that only in model membranes most closely resembling the plasma membrane of Gram-negative bacteria does the secondary structure of pleurocidin differ from that of magainin 2^17^. The present study therefore is concerned with testing whether MD simulations could faithfully reproduce such features of peptide-membrane interactions, where differences in the membrane active behavior and conformation have been observed between pleurocidin and magainin 2 in the steady state, and provide molecular level information that would explain why and/or how these structurally related peptides operate in such a different manner to kill Gram-negative bacteria and better understand the much greater potency of pleurocidin.

Specifically, we have performed a series of all atom simulations using a graphic processing unit (GPU) platform. Simulations of relatively large systems, comprising ~170,000 atoms and designed to mimic the inner membrane of Gram negative bacteria, were run for 100 ns in duplicate. These membranes consisted of 384 zwitterionic and 128 anionic lipids and were challenged with either one, four or eight molecules of each AMP placed in water giving a total of twelve simulations and over 1.2 μs of total sampling time. Extraction of the peptide secondary structures, location in the membrane and effects on local lipid acyl chain order enabled comparison with experimental data, obtained in the steady state, far-UV circular dichroism (CD) to monitor peptide conformation^17^ and solid-state nuclear magnetic resonance (NMR) methods to monitor peptide orientation^18^,^19^,^20^ and lipid acyl chain order^20^,^21^. Though comparing differing timescales, the MD simulations and experimental data showed good agreement and together reveal features of the membrane interaction of pleurocidin, in particular its conformational flexibility, that contribute to its greater potency when compared with magainin 2.

## Materials and Methods

### Peptides and lipids

Magainin 2 (GIGKFLHSAKKFGKAFVGEIMNS-NH_2_) and pleurocidin (GWGSFFKKAAHVGKHVGKAALTHYL-NH_2_) were purchased from Pepceuticals Ltd (Nottingham, UK) as desalted grade. Further HPLC purification was performed using acetonitrile/water gradients using either a Waters Symmetry™ C8, 5 μm, 7.8 × 100 mm column or a Waters SymmetryPrep™ C8, 7 μm, 19 × 300 mm column. The lipids 1,2-dimyristoyl-*sn*-glycero-3-phosphocholine (DMPC), 1,2-dimyristoyl-*sn*-glycero-3- phospho-(1′-*rac*-glycerol) (DMPG), 1-palmitoyl-2-oleoyl-*sn*-glycero-3-phospho-(1′-*rac*-glycerol) (POPG), 1-palmitoyl_d31_-2-oleoyl-*sn*-glycero-3-phospho-(1′-*rac*-glycerol) (POPG-d31) and 1-palmitoyl-2-oleoyl-*sn*-glycero-3-phosphoethanolamine (POPE) were obtained from Avanti Polar Lipids, Inc. (Alabaster, AL) and used without further purification. All other reagents were analytical grade or better.

### NMR structure determination

The NMR samples consisted of a 1 mM peptide solution also containing 100 mM deuterated sodium dodecyl sulphate (SDS-d_25_) with 5 mM Tris(hydroxymethyl-d_3_)-amino-d_2_-methane buffer at pH 7. 10% D_2_O containing trimethylsilyl propanoic acid (TSP) was added for the lock signal and as internal chemical shift reference. The temperature was kept constant at 310 K during the NMR experiments. NMR spectra were acquired on a Bruker Avance 500 MHz spectrometer (Bruker, Coventry, UK) equipped with a cryoprobe. Standard Bruker TOCSY and NOESY pulse sequences were used, with water suppression using a WATERGATE 3-9-19 sequence with gradients (mlevgpph19 and noesygpph19). The ^1^H 90 degree pulse was calibrated at 37.04 kHz. The TOCSY mixing time was 90 ms, and the mixing time for the NOESY spectra was set to 150 ms. The relaxation delay was 1 s. 2048 data points were recorded in the direct dimension, and either 256 or 512 data points in the indirect dimension. The spectra were processed using Bruker TOPSPIN. The free induction decay was multiplied by a shifted-sine^2^ window function. After Fourier transformation, the spectra were phase corrected, a baseline correction was applied, and spectra were calibrated to the TSP signal at 0 ppm.

Assignments were carried out with the Sparky software^22^ and structure calculations were done with ARIA software^23^, using NOE restraints of backbone protons and proline H_δ_ protons only, and no dihedral angle restraints. Restraints involving the sidechain atoms of tryptophan were removed because they were violated in every model during and after the structure calculation. The ARIA software was configured to use manual assignments and not allowed to change them. Proton frequency windows were set to 0.02 and 0.04 for the direct and indirect dimension, respectively. Upper and lower bound corrections were disabled. Structures were calculated using torsion angle MD, with the slow cooling protocol as published^24^, followed by refinement in water using default ARIA settings. One hundred structures were calculated in eight iterations, and the ten best structures were kept. Network anchoring was enabled during the first three iterations. Results were analyzed with AQUA and PROCHECK_NMR software^25^, and python scripts developed in our laboratory. An overview of all backbone NOESY contacts that were used in the structure calculations is available in the supplementary material. The presence of H_α_-H i − (i + 3) contacts and H-H i − (i + 1) contacts is usually taken as an indicator for α-helix conformation. Structures of pleurocidin and magainin 2 in SDS were deposited in the RCSB Protein Data Bank with accession codes of 2ls9 and 2lsa respectively.

### MD simulations

The simulations were run using the GPU package for simulation acceleration^26^,^27^,^28^ of LAMMPS (Large-scale Atomic/Molecular Massively Parallel Simulator), an open source molecular dynamics code distributed by Sandia National Laboratories at http://lammps.sandia.gov 29. The CHARMM force field^26^,^27^,^28^ was used to model the interactions of the counter ions, proteins^30^, and lipids^31^,^32^. The TIP3P water model^26^, which was modified for use with the CHARMM forcefield^28^, was used to describe interactions involving water. The lipid ratios (POPE/POPG 3:1) for the model membrane were chosen to reflect the lipid charge ratios of the inner membrane of Gram-negative bacteria. Since full folding of the peptides was not expected to be completed in the 100 ns simulations, the structures obtained from the NMR studies described above were used as the input for all atom MD simulations to determine whether the structural differences determined by CD in lipid bilayers, and which differed from that observed in SDS, could be explained. The conformation of the peptides was not constrained and modest unfolding of the peptide was expected before any penetration of the bilayer. All simulations described here are based around a lipid bilayer comprising 512 lipids, where 25% of the lipids were anionic and the remainder zwitterionic. The systems also contained 128 Na^+^ and 5 Cl^−^ ions and around 13,000 water molecules in addition to the either one, four or eight peptide molecules giving a total of approximately 170,000 atoms. The bilayer dimensions were 12 nm × 12 nm × 3.5 nm and the total system dimensions were 12 nm × 12 nm × 10 nm. Peptides were all placed in the water 2 nm above the same leaflet to simulate attack on the membrane from the outside only and a large water complement was chosen to prevent peptides crossing the periodic boundaries and approaching the bilayer from the other side, with the additional benefit of reducing the salt concentration. Histidine residues were positively charged following observations from our previous work^33^ that indicate anionic membranes support a substantially raised p*K*_a_, supported by experimental data monitoring the pH dependent disordering of POPG/POPG-d31 membranes by pleurocidin and magainin 2 (Supp. Fig. 1).

The starting conformations of the peptides were randomly oriented with respect to the bilayer, with a different orientation for the repeated simulation. The simulated system was prepared by first a series of NVE simulations, each of which were 1000 timesteps in duration, and the size of the timestep was increased in each consecutive simulation from 0.001 to 1.0 fs. During each of these simulations, position restraints were used to keep the position of each protein atom fixed and therefore only move the solvent atoms that were in close contact with the proteins. After these simulations, a NVT simulation was carried out for 1000 fs with a 1 fs timestep, and position restraints were placed only on the backbone atoms of the proteins, such that the side chain atoms could move but the NMR structure of the proteins would be conserved. Then this was followed with position restraints on every protein atom for 6 rounds of 1000 steps of 0.001–1.0 fs duration. This was followed by an NVT run of 1000 1.0 fs steps with position restraints on the protein backbone only, in order to conserve the NMR structures. This was followed by a NVT simulation utilising the Langevin thermostat to control the temperature at 310 K in which the time step was increased from 1.0 fs to 2.0 fs over the course of 20,000 timesteps. This process allows us to thermalise our system at the desired the temperature while using a slightly larger timestep. Hydrogen bonds and angles were constrained using the SHAKE algorithm^34^. The production simulations were run for 50,000,000 steps of 2.0 fs duration (100 ns) using the NPT ensemble with trajectories recorded after every 5,000 steps. The LAMMPS implementation^35^,^36^,^37^,^38^ of the Nose-Hoover thermostat^39^ was used to fix the system temperature in all production simulations, while the system pressure was controlled using a Nose-Hoover barostat^40^ in the NPT simulations. The trajectories were processed and analysed using code written in house. Programs were written in the Python programming language^41^, with specific use of the SciPy^42^, NumPy^43^,^44^, and matplotlib^45^ modules for scientific calculations, array analysis and graph plotting. The matplotlib python module was used to generate plots for the results. The Visual Molecular Dynamics (VMD) software was used to visualise the systems^46^.

### Liposome preparation

For solid-state NMR, samples with the lipid composition POPE/POPG-d31 (80:20, mol:mol) were prepared. A total of around 5 mg lipids per sample were dissolved and mixed in chloroform and dried under rotor-evaporation at room temperature. In order to remove all organic solvent, the lipid films were exposed to vacuum overnight. The films were then rehydrated with 4 ml of a suspension of magainin 2 or pleurocidin in 10 mM Tris/piperazine buffer at various pH at room temperature. Peptides were present at a final concentration, relative to the lipids, of 2% by mol. Samples were subjected to five rapid freeze-thaw cycles for further sample homogenization, generating multi-lamellar vesicles, and then centrifuged at 21000 g for 30 min at room temperature. The pellets, containing lipid vesicles and associated peptides were transferred to Bruker 4 mm MAS rotors for NMR measurements. Lipid vesicles were also prepared in this way in the absence of peptide. For circular dichroism experiments small unilamellar vesicle samples with different lipid compositions were prepared as above (molar ratios in brackets): DMPC/DMPG (75:25), and POPE/POPG (75:25). After rehydration with 5 ml of 5 mM Tris-amine buffer at pH 7.2 at room temperature, samples were sonicated on a Soniprep 150 (Measuring and Scientific Equipment, London, UK) for 3 × 7 minutes with amplitude of 6 microns.

### Solid-state ^2^H NMR

^2^H quadrupole echo experiments^47^ for samples containing POPG-d31 were performed at 61.46 MHz on a Bruker Avance 400 NMR spectrometer using a 4 mm MAS probe, spectral width of 100 KHz and with recycle delay, echo delay, acquisition time and 90° pulse lengths of 0.25 s, 100 μs, 2.6 ms and 3 μs respectively. The temperature was maintained at 310 K to keep the bilayers in their liquid-crystalline phase. During processing the first 10 points were removed in order to start Fourier-transformation at the beginning of the echo. Spectra were zero filled to 1 k points and 50 Hz exponential line-broadening was applied. Smoothed deuterium order parameter profiles were obtained from symmetrised and dePaked ^2^H-NMR powder spectra of POPG-d31 using published procedures^48^,^49^,^50^.

### Circular dichroism

Far-UV spectra were acquired on a Chirascan Plus spectrometer (Applied Photophysics, Leatherhead, UK). Liposome samples were maintained at 310 K. Spectra were recorded from 260 to 190 nm for liposomes composed of lipids with saturated acyl chains or from 260 to 200 nm when mono-unsaturated acyl chains were present. Lipid suspension was added to a 0.5 mm cuvette at a final concentration of 5.0 mM and then a few μl of a concentrated peptide solution were added and thoroughly mixed to give the indicated final peptide-to-lipid molar ratios. In processing, a spectrum of the peptide free suspension was subtracted and Savitsky-Golay smoothing with a convolution width of 5 points applied.

## Results

### NMR structure determination

**Figure 2:**
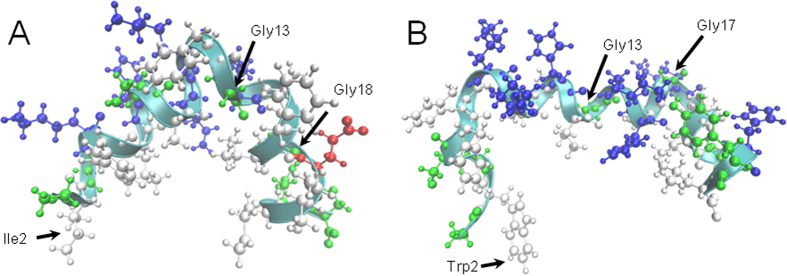
Cartoon representations of the two AMP structures. Structures were determined by ^1^H NOESY NMR spectroscopy in the presence of 100 mM SDS-d_25_ and used as the starting structures for the MD simulations. Key residues described in the text are shown for magainin 2 (**A**) and pleurocidin (**B**).

The NMR structures of pleurocidin and magainin 2 were determined in SDS in an attempt to replicate the anionic surface of bacterial membranes. Structures for both peptides have been determined previously in DPC micelles^51^,^52^ and SDS^51^,^53^ but only the structures determined for pleurocidin and magainin 2 in dodecylphosphocholine (DPC) micelles are available in the Protein Data Bank (1Z64 and 2MAG respectively). The structures of both peptides reveal a predominantly α-helical conformation, in agreement both with the previous NMR studies and far-UV CD spectra obtained in the corresponding environment^17^ (Fig. 2). For both peptides, an amphipathic distribution of polar and non-polar residues is maintained while considerable curvature of the helix long axis is observed which may be a result of the surface curvature of the SDS micelles. For both peptides conformational flexibility is manifested as distortions to the helix around Gly13 and either Gly18 or Gly17 for magainin 2 and pleurocidin respectively. Substitutions of glycine at either position 13 or 17, or both, in a series of pleurocidin analogues increased hemolytic activity but did not significantly affect antibacterial activity^54^. Interestingly only the double substitution leads to a substantial increase in α-helix content.

Taken together, these results suggest either that the structural features afforded by the glycine residues confer antibacterial selectivity (rather than potency) or that residues flanking the glycines may have a greater influence on conformation and antibacterial activity. Indeed, while pleurocidin has glycines at positions 1, 3, 13 and 17, magainin 2 has a very similar distribution with glycines at positions 1, 3, 13 and 18. Though resembling each other, there are notable differences in the NMR structures of magainin 2 and pleurocidin determined in SDS micelles. The curve or kink around Gly13 is very pronounced in the structure determined for magainin 2 in SDS in the present study (consistent with that detected in the structure of magainin 2 determined in DPC). For pleurocidin, the distortion around Gly13 is less pronounced but, additionally, a pronounced kink and distortion of the helix can be observed in the N-terminal helix that extends from Ser4 to Gly13.

This is consistent with the structural flexibility identified in both the N-terminal helix and the C-terminal helix that extends from Gly17 to Leu25 in the previous studies in both SDS^53^ and DPC^52^. In contrast with pleurocidin however, the helix distortion around Gly18 in magainin 2 is less pronounced when compared with that around Gly17 in pleurocidin suggesting magainin 2 may lack some structural flexibility in this and the N-terminal region when compared with pleurocidin.

### Secondary structure analysis in membranes

**Figure 3:**
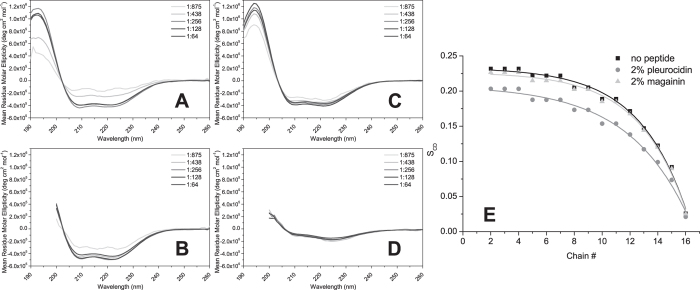
Secondary structure and membrane disordering in the steady state. Dose dependent changes in far-UV CD spectra characterize the secondary structure of magainin 2 (**A**,**B**) or pleurocidin (**C**,**D**) in the presence of 5 mM DMPC/DMPG (75:25) (**A**,**C**) or POPE/POPG (75:25) (**B**,**D**) - pleurocidin or magainin 2 are present at the indicated molar ratios relative to the combined lipid fractions. Smoothed order parameter profile (**E**) obtained from solid echo ^2^H solid-state NMR spectra of multi lamellar vesicles comprising POPE/POPG-d31 (80:20) and 2 mol% pleurocidin or magainin 2 in 20 mM Tris-amine pH 7.3.

In duplicate runs, both pleurocidin and magainin 2, whether administered individually or as a group of four or eight peptides, were observed to insert into the interface region of the membranes, between the top leaflet and water phase. However, substantial differences particularly in secondary structure, depth of membrane penetration and topology were observed. The secondary structure of cationic amphipathic AMPs, in particular the ability to adopt α-helix conformation in membranes, has long been considered a key determinant of antibacterial potency^55^ and, though some have proposed the ability to reside in the interfacial region is sufficient for antibacterial activity, disruption of α-helix can cause substantial reductions in antibacterial potency towards Gram-negative bacteria^56^. In our previous study we used CD spectroscopy to experimentally determine the secondary structure of pleurocidin and magainin 2 in a variety of traditional membrane mimicking environments and lipid vesicles^17^. Both peptides adopt near identical α-helix conformations in 50% TFE, 50 mM SDS or, when added at 0.5 mol%, lipid vesicles comprising either DMPC/DMPG (80:20) or POPC/POPG (80:20)^17^. Only in POPE/POPG (80:20) liposomes were any differences in secondary structure apparent where the α-helix content of pleurocidin was notably reduced. Here we extend our CD studies to monitor the conformation of magainin 2 and pleurocidin over a range of peptide to lipid ratios to provide more appropriate comparisons with the simulations (Fig. 3). The far-UV CD spectra confirm that only in POPE/POPG (75:25) is the α-helix content of pleurocidin reduced c.f. DMPC/DMPG (Fig. 3C,D) and that this occurs irrespective of peptide concentration. Magainin 2 adopts α-helix conformation at the peptide:lipid ratios used in the simulations but there is some evidence of a dose dependency at low peptide concentrations (Fig. 3A,B).

**Figure 4:**
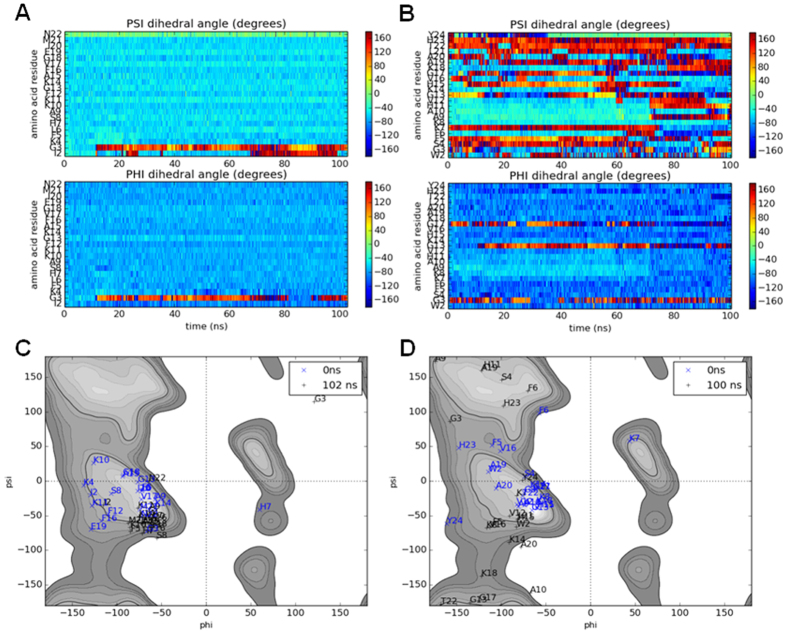
Conformational flexibility of pleurocidin is replicated in MD simulations. Secondary structure analysis of the binding of a single magainin 2 (**A**,**C**) or pleurocidin (**B**,**D**) molecule to membranes consisting of 128 POPG and 384 POPE lipids. Ramachandran plots for snapshots obtained at the start and end of 100 ns runs are compared (**C**,**D**) while phi and psi angles for individual peptide residues are also plotted as a function of time (**A**,**B**).

If simulations of the initial binding of the peptides to membranes, run for 100 ns, can be effectively related to experimental data obtained in the steady state, we should expect the α-helix conformation of magainin 2 to be largely preserved throughout the simulations while that of pleurocidin might reduce over the course of the simulation. The secondary structures of a single magainin 2 or pleurocidin molecule, incubated in the presence of POPE/POPG membranes, are compared in Ramachandran plots for the starting configuration, structures determined in SDS, and late snapshots (Fig. 4C,D) and by comparing phi and, psi angles as a function of time (Fig. 4A,B). The Ramachandran plot for magainin 2 shows almost all residues concentrated in the region expected for α-helix (phi between −90° and −35°, psi between −70° and −15°) at both the start and end of the 100 ns run, indeed the distribution of phi and psi angles is much lower after 100 ns in contact with the POPE/POPG membrane (Fig. 4C).

In contrast, the Ramachandran plot for pleurocidin shows that much of the α-helix content of the peptide has been lost over the course of the simulation (Fig. 4D); at the start of the run most of the residues are concentrated in the α-helix region, albeit with a number distributed in other regions, but after 100 ns in the presence of POPE/POPG, a much greater distribution of, particularly, psi angles can be seen indicating that much of the α-helical secondary structure has been lost. When compared over time, the dynamic change in secondary structure is evident. For magainin 2, little or no deviation from the starting α-helix conformation is noted over the course of the simulation (Fig. 4A) except in the immediate vicinity of the N-terminus. In contrast, for pleurocidin (Fig. 4B), a rapid loss of α-helix content is observed at the N-terminus and between Gly13 and Tyr24. The α-helix conformation of residues Lys8 to Val12 persists for around 70 ns but is ultimately lost and after 100 ns the majority of the peptide is disordered. This qualitative difference is a feature of duplicate simulations conducted with either one, four or eight pleurocidin or magainin 2 molecules (Supp. Figs 2–8). Comparison of secondary structure changes at higher peptide to lipid ratios is achieved by averaging psi and phi over all eight peptides during the 100 ns simulations (Supp. Fig. 8). This comparison confirms the observation that the region from Lys8 to Val12 in pleurocidin has, on average, lower conformational disorder and indicates that in the C-terminal segment an ordered region (Ala19-His23) is flanked by disordered segments around Gly17 and at the C-terminus itself. Although some conformational disorder is also induced in magainin 2, the much greater conformational flexibility of pleurocidin is evident with substantial fluctuation in psi angles throughout the peptide (Supp. Fig. 8E,F). These findings are consistent with the CD data obtained in the steady state (Fig. 3).

Our analysis of secondary structure changes in the peptides includes the Ramachandran plots, circular variance of phi and psi angles and also an analysis of hydrogen bonding using the improved DSSP program^57^ (Supp. Figs 6 and 7). The new version of this software strictly follows the original description of secondary structure^58^, assigning π-helices before α-helices. While the vast majority of residues in the eight pleurocidin peptides adopt bend or “other” conformations the conformation of residues in the majority of magainin 2 peptides that remain ordered is assigned as π-helix rather than α-helix. This was qualitatively replicated in the duplicate simulations. The Ramachandran analysis is consistent with both these helix conformations since the sum of phi and psi fall in the same range. Sections of π-helix in proteins are typically no more than ten residues and hence, to our knowledge, CD is not yet able to distinguish between π-helix and α-helix. Consequently, we are unable to conclude whether magainin 2 indeed adopts a stable π-helix when binding to POPE/POPG membranes in preference to the more common α-helix conformation.

### Conformation, orientation and depth of penetration

**Figure 5:**
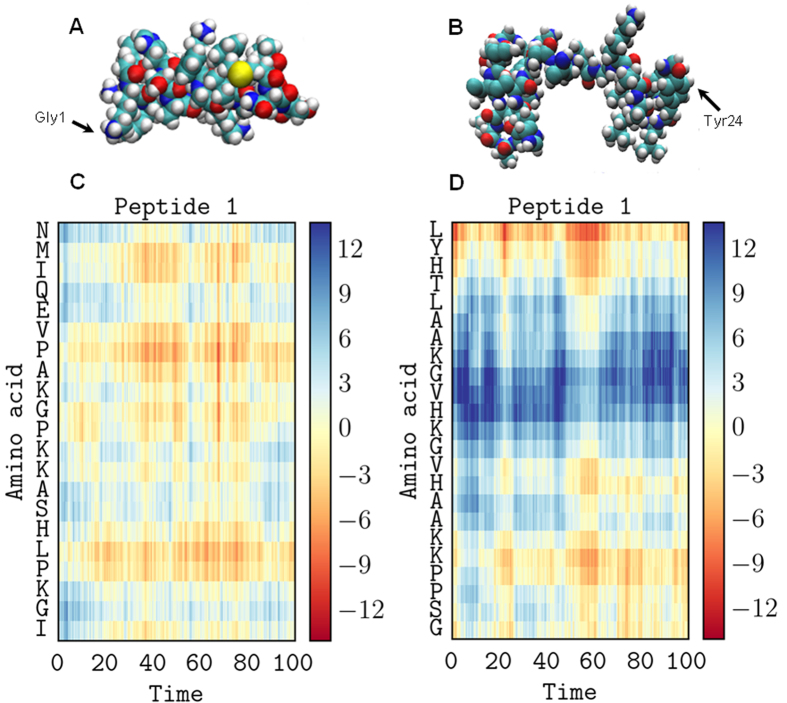
Conformational flexibility and alignment. Space-filling models of magainin 2 (**A**) and pleurocidin (**B**) showing representative conformations in the membrane of one of eight peptides in each simulation at 100 ns. The distance (Å) to the upper membrane leaflet phosphate plane of each amino acid in the representative peptide is plotted over 100 ns for magainin 2 (**C**) and pleurocidin (**D**).

The ability to determine the orientation of peptides with respect to the membrane is a key strength of solid-state NMR. This parameter has been measured for both magainin 2 and pleurocidin in POPE/POPG membranes using solid-state ^15^N NMR of aligned samples peptides incorporating ^15^N labelled amino acids at specific sites^18^,^19^,^20^. For pleurocidin^20^, a single broad resonance at 87 ppm was observed for samples containing ^15^N labels at Ala10, Ala19 and Leu21 while for magainin 2, separate samples were prepared with labels incorporated at a range of positions between Ile2 and Ile20. The observed ^15^N chemical shifts, between 42 and 57 ppm, and ^1^H-^15^N dipolar splitting were consistent with a surface, rather than a transmembrane, aligned orientation for magainin 2. The value obtained for the analogously labeled pleurocidin is notably downfield of those reported for magainin 2 but both peptides are predicted to be aligned approximately parallel (as opposed to perpendicular) with the membrane surface. The prediction of alignment based on solid-state NMR of such peptides is based on the assumption of a relatively uniform α-helix conformation throughout the length of the peptide. Since possible intermolecular (peptide) interactions (described below) may impact on conformation and alignment simulations comprising eight peptides are used for comparison with experiment (Fig. 5; Supp. Figs 9 and 10). The disorder in the secondary structure of pleurocidin in POPE/POPG membranes (Fig. 5F) facilitates a conspicuously bent configuration as shown in the final snapshot of one, representative peptide from the duplicate simulations (Fig. 5B). This bent configuration is consistent with the downfield shift of the ^15^N chemical shift assigned to the three labeled sites in pleurocidin^20^ when compared with those obtained for magainin 2^18^,^19^. The MD simulations now reveal why the conformational flexibility between residues Gly13 and Tyr24 and also at the N-terminus is important; a pronounced kink forms between Gly13 and Lys18 ensuring hydrophobic residues at the N- and C-termini (Phe5, Phe6, Ala19, Ala20, Leu21) are oriented to plunge further into the hydrophobic core of the membrane (Fig. 5D). In contrast, magainin 2, though occasionally displaying more conformational disorder when eight rather than a single peptide challenge the POPE/POPG membrane, retains a compact configuration while residing in the membrane interface region (Fig. 5A), with all residues approximately equidistant from the bilayer phosphate plane (Fig. 5C). Furthermore, conformational disorder, observed between Ala8 and Gly13, does not extend into the C-terminus of the peptide, despite the presence of an additional glycine (Gly18) in this region.

**Figure 6:**
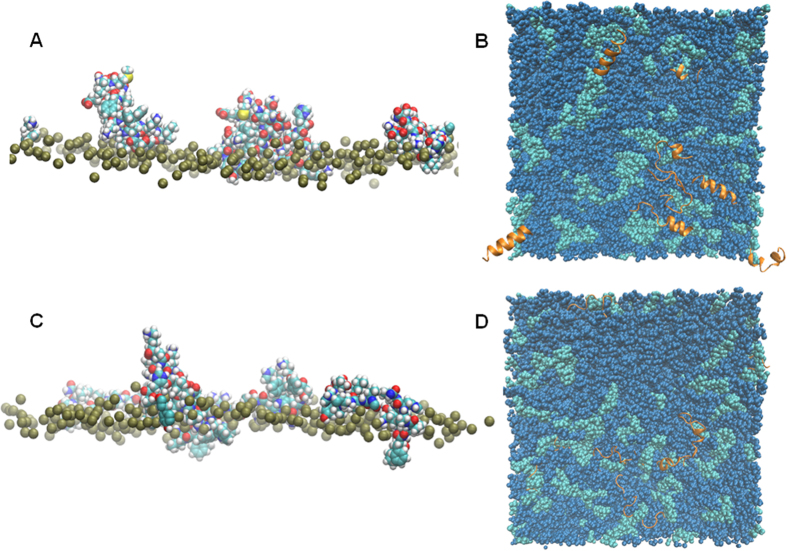
Penetration and aggregation. Side view of the magainin 2 (**A**) and pleurocidin (**C**) four peptide simulations at 100 ns. For clarity, phosphate atoms (gold spheres) are shown for lipids in the top leaflet only. Top view snapshots of simulations for magainin 2 (**B**) and pleurocidin (**D**) of eight peptides in POPE/POPG membranes at 100 ns. The results show clustering of the POPG (green) lipids around the peptides, self-association of magainin 2 as well as a substantial loss of ordered helix conformation for pleurocidin (**B**).

The effect of the increase in conformational disorder and bent configuration observed for pleurocidin are substantial. When four peptides are used to challenge the membrane, a much greater penetration of pleurocidin into the POPE/POPG membranes is apparent when compared with magainin 2 (Fig. 6A,C). Also notable, when the behavior of eight peptides is simulated, is a greater self-association of magainin 2 at the membrane surface (Fig. 6B) compared with pleurocidin (Fig. 6D). This self-association sees four of the eight magainin 2 molecules in close contact. Similar behavior was observed for the duplicated simulation of eight magainin 2 molecules but not in either of the corresponding simulations for eight pleurocidin molecules; this difference is notably reflected in the much greater number of Cα to Cα contacts between residues in distinct peptides that are <10 Å in both magainin 2 simulations (Supp. Fig. 11). Both peptides have a slight preference to be associated with the anionic POPG over the zwitterionic POPE though the extent of this preference for the negatively charged component is dependent on the number and type of peptide in the simulation. This is quantifiable by plotting the number of lipid atoms within 4 Å of the peptide (Supp. Fig. 12). Comparing the insertion of four magainin 2 or pleurocidin molecules, it is clear that a much larger number of lipid atoms come into contact with pleurocidin than magainin 2 by the end of the 100 ns simulation (Supp. Fig. 12A). Both peptides have a preference for POPG over POPE as the ratio of POPE to POPG lipids encountered, on average, by the four peptides deviates substantially from the expected value of 3 (Supp. Fig. 12C). However, when eight peptides are used to challenge the membrane the difference between pleurocidin and magainin 2 is less apparent as both peptides encounter similar numbers of both types of lipids (Supp. Fig. 12B) although pleurocidin has a somewhat greater preference for the anionic POPG than magainin 2 (Supp. Fig. 12D). These observations are an apparent manifestation of a concentration dependent membrane insertion by magainin 2, which contrasts with the concentration independent insertion of pleurocidin. These findings are again consistent with the CD data obtained in the steady state (Fig. 3).

### Membrane disorder

The concentration dependent differences in the ability of magainin 2 and pleurocidin to insert and preferentially associate with lipids of a particular type may be reflected in their ability to induce disorder in the acyl chains of lipids in their immediate vicinity. Both experimentally^20^,^21^ and in previous MD simulations^9^,^10^, cationic α-helical AMPs have been shown to induce lipid disorder in lipid bilayers. In particular in simulations, for the leaflet where magainin H2 bound, a greater disordering effect was observed for those lipids associated with the peptide while those lipids that were not associated became more ordered when compared with the peptide free membrane^9^. The bilayer as a whole saw a marginal increase in order when challenged with the peptide. Experimentally, pleurocidin has been observed to have a greater lipid disordering effect on the anionic lipid component when compared with the zwitterionic component^20^. The disordering effect of pleurocidin and its inhibition has been linked to the ability/inability to from pores and occasion dye release from similarly prepared liposomes^21^.

**Figure 7:**
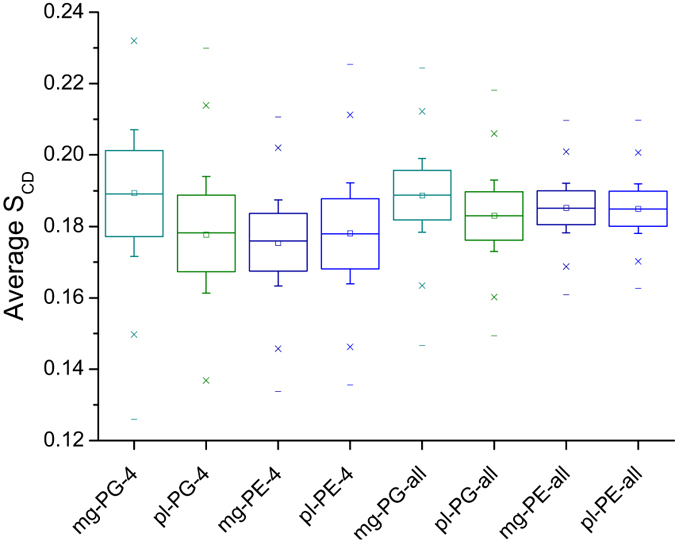
Disordering of individual lipid components. Average order parameters (last 50 ns) of magainin 2 (**mg**) and pleurocidin (**pl**) eight peptide simulations for all lipids (-**all**) or those lipids within 4 Angstroms (**-4**) of any peptide. Box – 25–75% of data, whisker - ± one standard deviation. Statistical comparisons described in the text satisfy (*p* < 0.05) Wilcoxon Signed Ranks Test, Paired Sample t Test and OneWay ANOVA.

In the present study we investigated whether the membranes constructed and equilibrated *in silico* corresponded to analogous membranes studied *in vitro*. Smoothed order parameters (S_CD_) were obtained from solid-echo ^2^H NMR spectra of multilamellar vesicles, maintained in aqueous suspensions at 310 K, containing mixtures of POPE and POPG with either POPG-d31 (Fig. 2E) or POPE-d31 (not shown) as a reporter. When the smoothed order parameter profiles are averaged over the length of the acyl chain, S_CD_ is 0.179 and 0.177 for the anionic and zwitterionic components respectively. These can be compared with calculated order parameter profiles obtained from the simulations for all lipids or restricted to those within a 4 Å radius of the peptide (Fig. 7; Supp. Fig. 11). The area per lipid and membrane thickness do not differ substantially over time and show little, if any, effect of challenge with even eight peptides (Supp. Fig. 12). For the ensemble of lipids, the average order parameters, sampled over the last 50 ns of each simulation, are consistent with the experimentally determined value, perhaps revealing a slight increase in order for lipids distant from the peptides. The localized disordering effect of both peptides, within a 4 Å radius of the peptide, reveal a modest but significant increase in disorder, relative to the ensemble of lipids, of both POPG and POPE, by pleurocidin, and POPG, but particularly, POPE by magainin 2 (Fig. 7). Pleurocidin therefore has a significantly greater disordering effect on POPG when compared with magainin 2 with the opposite observed for POPE (Fig. 7). Consequently, disordering of POPE and POPG in the presence of eight pleurocidin molecules is not significantly different but magainin 2 disorders POPE lipids significantly more than POPG (Fig. 7). However, even though this peptide induced lipid acyl chain disordering is detectable, it is modest even when eight peptides are used to challenge the membrane and longer timescales (300 ns–1 μs) may be required to see more substantial differences. For comparison the average S_CD_ for POPG-d31 challenged with 2 mol% peptide in the steady state are 0.151 and 0.176 for pleurocidin and magainin 2 respectively.

## Discussion

The present study originates in the observation that two seemingly similar antimicrobial peptides can share a large number of physical and structural properties but can have dramatically different potency against the same bacteria^17^. Pleurocidin and magainin 2 are of similar length, hydrophobicity, hydrophobic moment (when adopting an ideal α-helix conformation) and charge^17^. Using a combined systems approach we have recently shown that changes in gene expression, combined with analysis of the gene ontology can reveal, in particular, which cellular components are affected by AMP challenge which, in turn reveals considerable insights into the mechanism of action of the AMP^4^. The study indicates that, for *E. coli*, magainin 2 acts predominantly on the cell envelope while pleurocidin affects both the cell envelope and also intracellular components. This finding is consistent with the observations that pleurocidin inhibits macromolecular synthesis in *E. coli*, when administered at sub-lethal concentrations^59^, and has a greater affinity for nucleic acids when compared with magainin 2^17^. Pleurocidin demonstrates a 10-fold improvement in antibacterial potency over magainin 2 against *E. coli* (NCTC 9001) and *P. aeruginosa* (PAO1). When tested against *E. coli* (TOP10), a competent strain similar to DH10B which is deficient in *galU, galK* and *galE* and hence expected to have an altered lipopolysaccharide structure^60^, both peptides had much improved potency but the 10-fold difference in potency was maintained^17^. Since the outer membrane of Gram-negative bacteria is considered a poor barrier to AMPs, this indicates that the differences in potency are most likely determined by, or downstream of, their interaction with the inner membrane. Pleurocidin and magainin 2 adopt near identical α-helix conformations in a variety of traditional membrane mimicking media and model membranes^17^. However a difference in conformation was determined in a POPE/POPG membrane which, interestingly, is the closest of the models used^17^ to the inner membrane of Gram-negative bacteria. We therefore chose to investigate the conformation and behavior of pleurocidin and magainin 2 in POPE/POPG membranes as a model of the inner membrane of Gram negative bacteria in order to determine which features and strategies promote antibacterial potency in cationic α-helical AMPs.

Since the magainin antibiotic peptides were described 25 years ago^61^, a considerable body of work has focused on understanding the mechanism of action of this peptide with a view to engineering a replacement for classical antibiotics which are struggling in the face of the emergence of resistant bacteria. The interaction of magainin 2 with lipid bilayers has been assessed using both a large number and variety of biophysical methods. At lipid to peptide ratios above 20:1 there is broad agreement^19^,^62^,^63^,^64^ that magainin 2 adopts an alignment roughly parallel to the surface of the membrane. Oriented circular dichroism measurements at lipid to peptide ratios below 20:1 then indicate that some peptide adopts an orientation perpendicular to the membrane surface^65^ and neutron in-plane scattering measurements detect toroidal shaped pores^62^. Though the observed pores have formed the cornerstone of many, if not all, models for the action of cationic α-helical AMPs^8^ there is some unease that such high concentrations of peptide are required for such pores to be observed as this does not leave much scope for differences in peptide potency to be explained. Furthermore, the studies described above were performed in a variety of anionic and, sometimes, zwitterionic lipid membranes and magainin 2 has been observed to behave somewhat differently according to the membrane lipid composition; causing either graded or all-or-nothing dye release from liposomes at lipid to peptide ratios as high as 200:1 with the former linked to peptide translocating across the membrane^66^. In earlier MD simulations, magainin-H2 has been shown to form disordered toroidal pores in zwitterionic membranes and translocate to the internal leaflet at lipid to peptide ratios of between 64:1 and 32:1^9^. Finally, though magainin 2 is considered the archetypal pore forming AMP, the observation that for at least one microbial target, *Saccharomyces cerevisiae*, magainin 2 is capable of entering the cell to interfere with DNA integrity^67^ suggests a more detailed understanding of peptide membrane interactions is required to understand the wider effects of magainin 2 on its targets as well as the key determinants of potency.

A number of synthetic peptides with increased potency have been derived from magainin 2, most notably MSI-78 (also known as pexiganan). This peptide retains the glycine residues in positions 1, 3 and 13 but not position 18 and has a dramatically increased nominal positive charge of +10 in its amidated form^68^. Increased conformational stability of MSI-78, compared with magainin 2, has been observed in 20 ns simulations in POPC membranes^69^ and this may suggest that increased α-helix content might correlate with increased anti-bacterial potency, as has been observed experimentally^55^,^70^. The improved antibacterial activity of MSI-78 compared with its parent peptide is likely therefore to be due to its increased cationicity and/or conformational stability. This might conceivably be the case for pleurocidin; if it is assumed that the histidine residues in both magainin 2 and pleurocidin are protonated when bound to anionic membranes, pleurocidin does have a notably greater cationicity with a nominal positive charge of +8 compared with the +5 of magainin 2. However, both experiment^17^ and the present simulations show that pleurocidin has substantially reduced α-helix content in the membranes that most closely mimic those of the targeted Gram-negative bacteria inner membrane. This is consistent with previous work by us^71^ and others^72^,^73^ that has shown that conformational flexibility, induced by proline kinks, and hence reduced α-helix conformation can often be beneficial for antibacterial potency.

The present study offers an interpretation of this observation by showing that the increased conformational flexibility of pleurocidin translates into a substantially altered configuration which enables much deeper penetration into the membrane at lower peptide to lipid rations when compared with its rival. Indeed, while for pleurocidin effective penetration into the membrane interface proceeds independently of concentration, only at higher peptide concentrations does magainin 2 penetrate into the membrane and this requires, or is at least correlated with, substantial peptide self-association in the interfacial region. The present model therefore suggests means by which the conformational flexibility of pleurocidin and its ability to penetrate membranes at lower peptide concentrations may increase its antibacterial potency by influencing both pore-forming and non-pore-forming bactericidal activities. However, further studies are required to better understand how this translates into the differing bactericidal behaviors. The role of α-helix conformation in the stabilization of disordered toroidal pores has been studied previously^74^. By restricting the α-helix content of magainin-H2 to defined values, it was shown that partial α-helix conformations were required for stable pores to form with high α-helix content causing pores to close on a microsecond timescale. This implies that the more disordered conformation of pleurocidin, compared with magainin 2 observed in POPE/POPG membranes in the present study will promote stable pore formation. In previous MD simulations focusing on magainins, pores were typically observed in zwitterionic membranes after between 17 and 63 ns at lipid to peptide ratios of 32:1 (and once at 64:1 when a lateral tension of 20 mN/m was applied)^9^, Over the 100 ns duration of our simulations in mixed zwitterionic/anionic membranes at lipid to peptide ratios of up to 64:1, no pore formation was observed for either magainin 2 or pleurocidin. The present study is therefore limited in its focus in attempting to identify differences between pleurocidin and magainin 2 at relatively short timescales and pore forming events cannot be ruled out at longer timescales or in the presence of a proton motive force, divalent cations or other membrane components such as cardiolipin. Furthermore such a short window may not be sufficient for complete lateral reorganization of the membrane, clustering of distinct lipid species and resultant lipid acyl chain disordering. This is an important limitation of the present study since, when longer timescales are explored using coarse graining techniques, AMPs have been shown to induce the growth of phosphatidylglycerol (PG) domains in mixed membranes^14^. PG plays an important role in maintaining the stability of bacterial membranes, limiting the protrusion of phosphatidylethanolamine (PE) lipid headgroups into the water phase and their motion along the bilayer normal^16^ and domain formation would be expected to have deleterious effects on membrane integrity. Indeed, the formation of PG rich domains has been linked experimentally to anti-bacterial action, particularly for bacteria whose membranes comprise a mixture of anionic and zwitterionic lipids such as Gram negative bacteria^75^. It has been further shown that a greater positive charge density confers the ability to cluster anionic lipids and that magainin 2 was not capable of promoting such clustering^76^. Future MD simulations should be extended to explore longer timescales to investigate whether and how pleurocidin may promote domain formation, and investigate whether greater penetration of a highly cationic molecule into the bacterial membrane may compromise the membrane potential and pH and electrochemical gradients by promoting electroporation, non-lytic membrane depolarization or by functioning as an anion carrier; ion channel activity has been observed experimentally for pleurocidin, linked to the formation of a toroidal pore^77^.

## Conclusion

The important role of subtle differences in peptide primary sequence suggests a greater level of sophistication can be fruitfully applied to structure - activity relationships designed to understand the multifaceted mechanism of action of AMPs. The relationship between antibacterial potency and dynamic peptide behavior in membranes closely matching the target cell membranes is apparent and provides a detailed understanding of biophysical data obtained in the steady state. The present study suggests the conformational flexibility of cationic AMPs is an important feature that confers anti-bacterial potency by facilitating access to the bacterial cytoplasm, enabling a wider variety of bactericidal effects.

## Additional Information

**How to cite this article**: Amos, S.-B. T. A. *et al*. Antimicrobial Peptide Potency is Facilitated by Greater Conformational Flexibility when Binding to Gram-negative Bacterial Inner Membranes. *Sci. Rep.* **6**, 37639; doi: 10.1038/srep37639 (2016).

**Publisher’s note:** Springer Nature remains neutral with regard to jurisdictional claims in published maps and institutional affiliations.

## Supplementary Material

Supplementary Figures

## Figures and Tables

**Figure 1:**
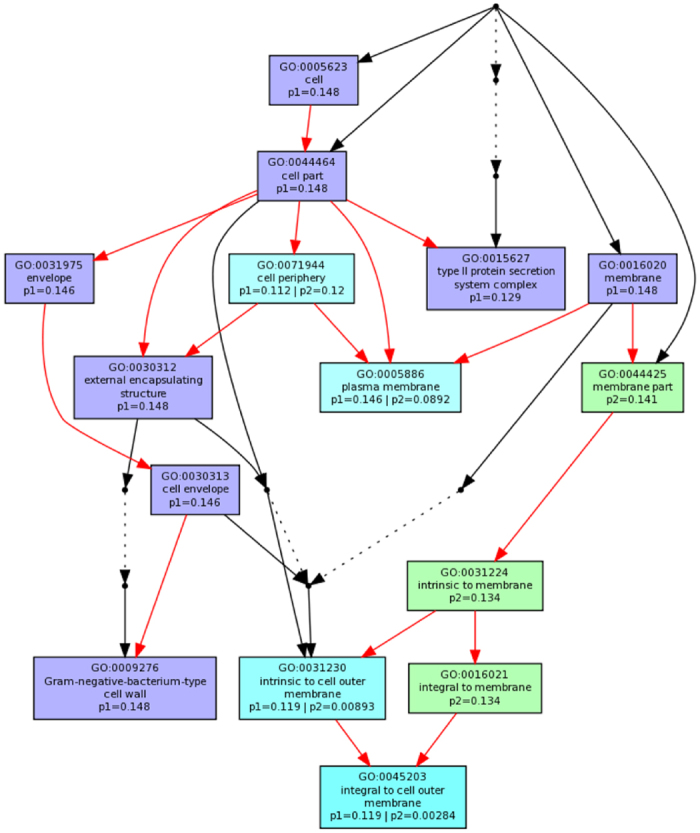
Transcriptome view of AMP mode of action. Multi GOEAST comparison of gene ontology (GO) terms relating to cellular component for the 200–250 most differentially expressed genes in *E. coli* NCTC 9001 induced by challenge with sub-lethal concentrations of pleurocidin (blue: p1) or magainin 2 (green: p2). Magainin 2 predominantly affects membrane components while pleurocidin has wider affects within the cell in addition to those detected at the plasma and outer membranes. Red arrows represent relationships between two enriched GO terms, black arrows between enriched and un-enriched terms and black dashed arrows represent relationships between two un-enriched GO terms. Raw *p* values for GO terms have been adjusted using the Benjamini-Hochberg method allowing FDR < 15%. Figure generated from previously reported data^4^.
