# Author Correction: Antimicrobial Peptide Potency is Facilitated by Greater Conformational Flexibility when Binding to Gram-negative Bacterial Inner Membranes

**DOI:** 10.1038/s41598-018-35519-9

**Published:** 2018-11-19

**Authors:** Sarah-Beth T. A. Amos, Louic S. Vermeer, Philip M. Ferguson, Justyna Kozlowska, Matthew Davy, Tam T. Bui, Alex F. Drake, Christian D. Lorenz, A. James Mason

**Affiliations:** 1 Institute of Pharmaceutical Science, King’s College London, Franklin-Wilkins Building, 150 Stamford Street, London, SE1 9NH United Kingdom; 2 Department of Physics, King’s College London, London, WC2R 2LS United Kingdom; 3 Present Address: Department of Biochemistry, University of Oxford, South Parks Road, Oxford, OX1 3QU United Kingdom; 4 Present Address: Biophysique des Membranes et RMN, Institut de Chimie (UMR-7177), 1 rue Blaise Pascal, Université de Strasbourg, Strasbourg, France

Correction to: *Scientific Reports* 10.1038/srep37639, published online 22 November 2016

This Article contains errors in the C and D panels of Figure 5 and in Supplementary Figures S9 and S10 where mislabelling occurred. The correct Figures 5, S9 and S10 appear below as Figures 1, 2 and 3 respectively.

**Figure 1:**
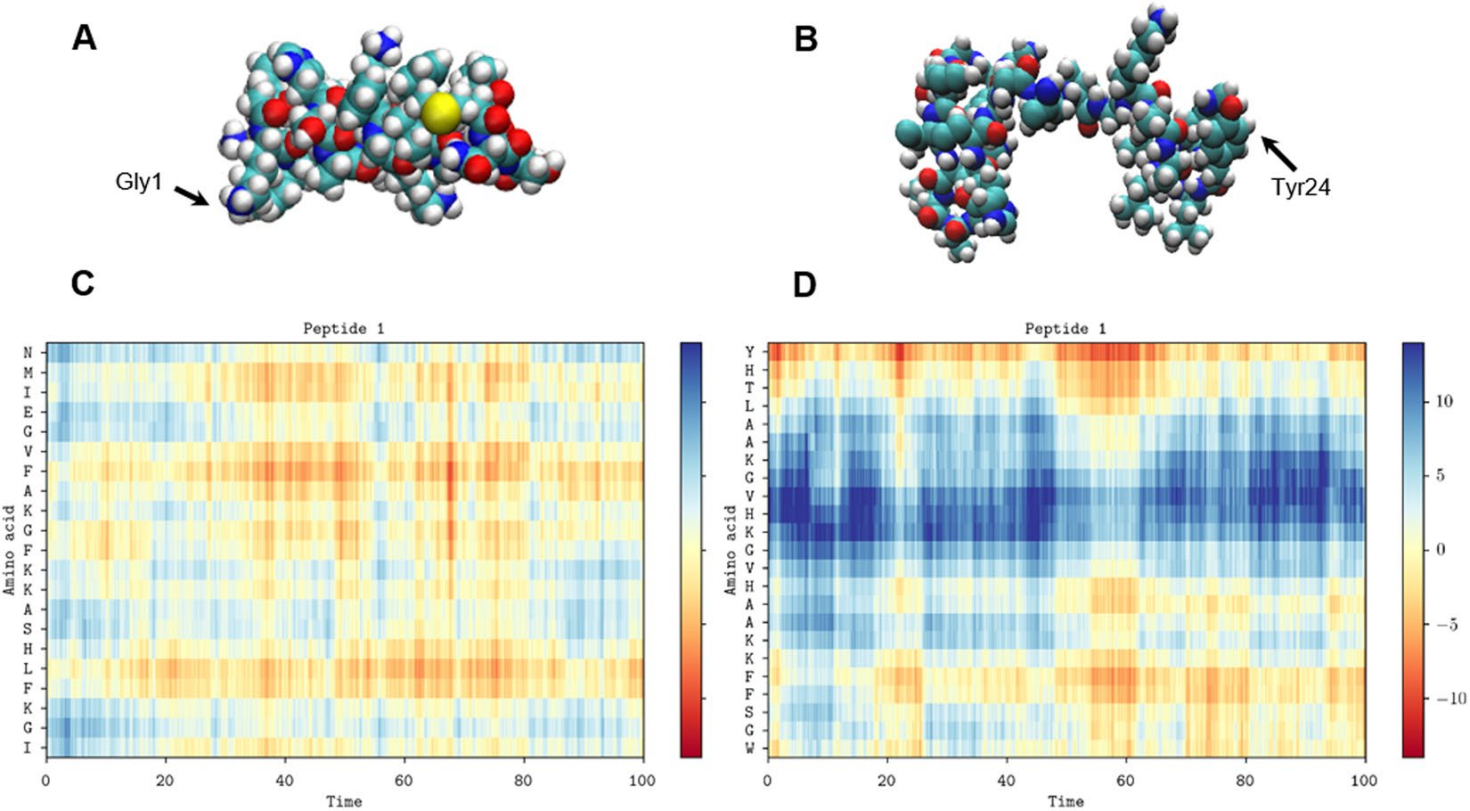
Conformational flexibility and alignment. Space-filling models of magainin 2 (**A**) and pleurocidin (**B**) showing representative conformations in the membrane of one of eight peptides in each simulation at 100 ns. The distance (Å) to the upper membrane leaflet phosphate plane of each amino acid in the representative peptide is plotted over 100 ns for magainin 2 (**C**) and pleurocidin (**D**).

**Figure 2:**
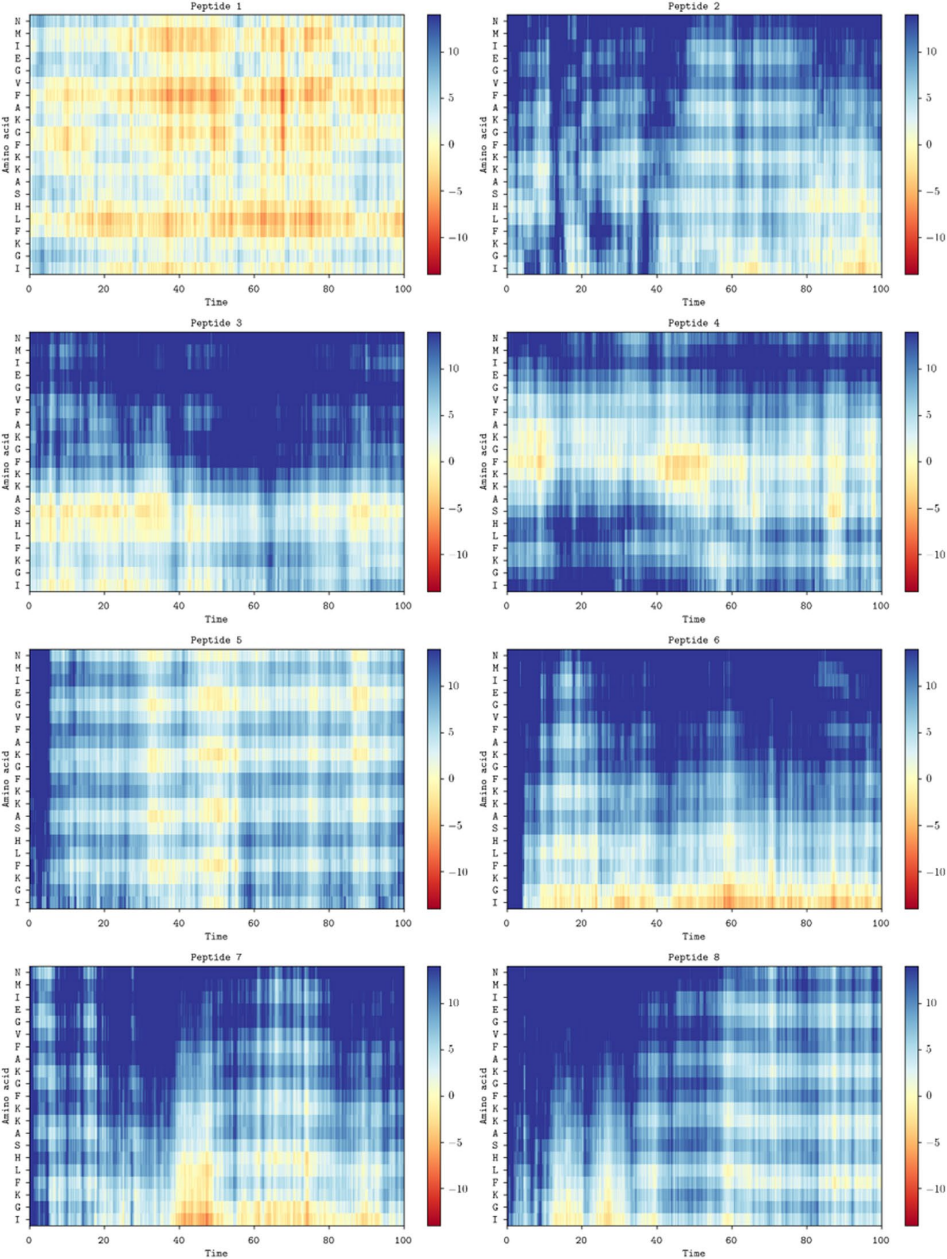
.

**Figure 3:**
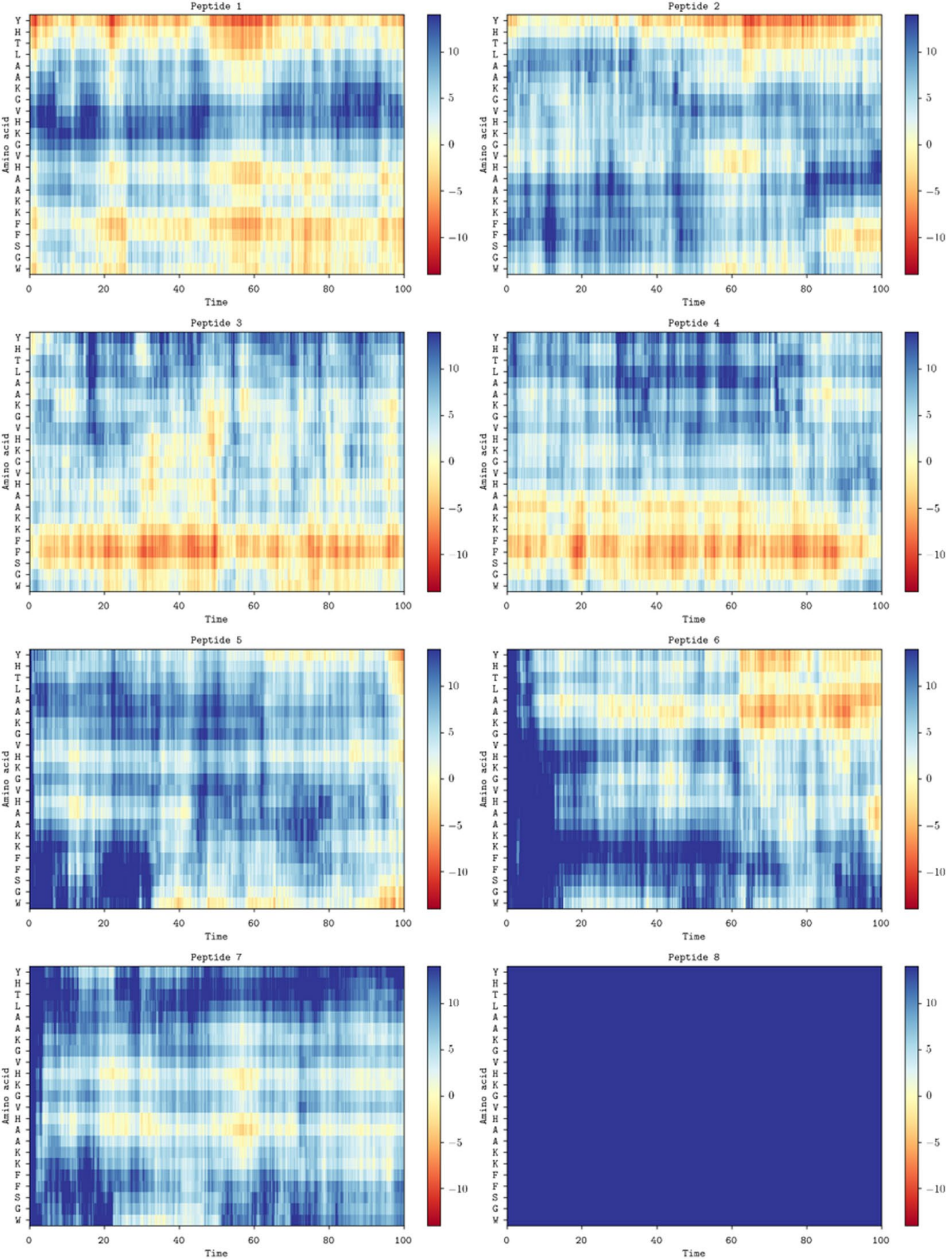
.

