# Supplementary material for: Antimicrobial Peptide Potency is Facilitated by Greater Conformational Flexibility when Binding to Gram-negative Bacterial Inner Membranes
Source: Sci Rep. 2016 Nov 22;6:37639. doi: 10.1038/srep37639 (PMC5118786; doi:10.1038/srep37639)
Supplement: Supplementary Figures [file srep37639-s1.pdf]

Antimicrobial Peptide Potency is Facilitated by Greater Conformational Flexibility when Binding to Gram-negative Bacterial Inner Membranes

Sarah-Beth T.A. Amos, Louic S. Vermeer, Philip M. Ferguson, Justyna Kozlowska, Matthew Davy, Tam T. Bui, Alex F. Drake, Christian D. Lorenz and A. James mason

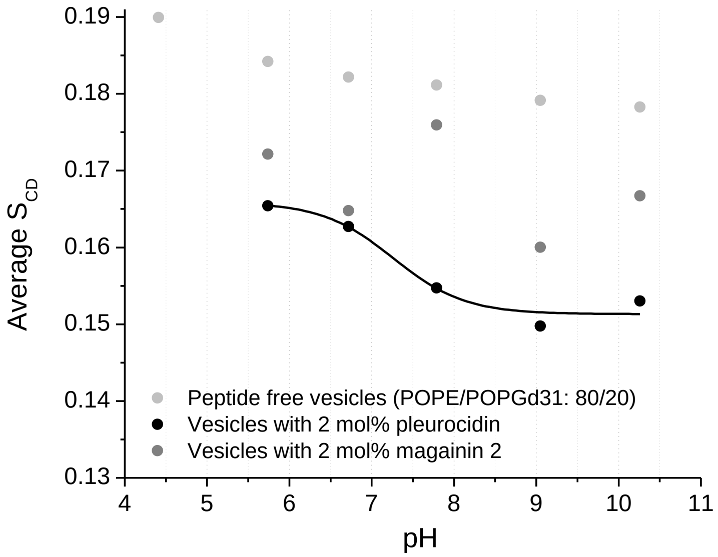

Sup. Fig. 1 pH dependent peptide induced membrane disordering. In the absence of peptide, a small increase in membrane order is observed at low pH which may be related to protonation of POPG. Disordering due to pleurocidin is pH dependent and is greater at basic pH. The pK_{mem} (the midpoint of the pH dependent change in membrane disordering) is 7.26 ± 0.31 and hence histidine residues were considered to be partially protonated in the presence of anionic membranes and a positive charge was conferred on these residues in the simulation starting configurations.

For magainin 2, much greater variation in peptide induced disordering was observed, which was always less than that induced by comparable amounts of pleurocidin. For consistency, His7 in magainin 2 was given a positive charge but, without conclusive support for its possible protonation, Glu19 was left with a negative charge.

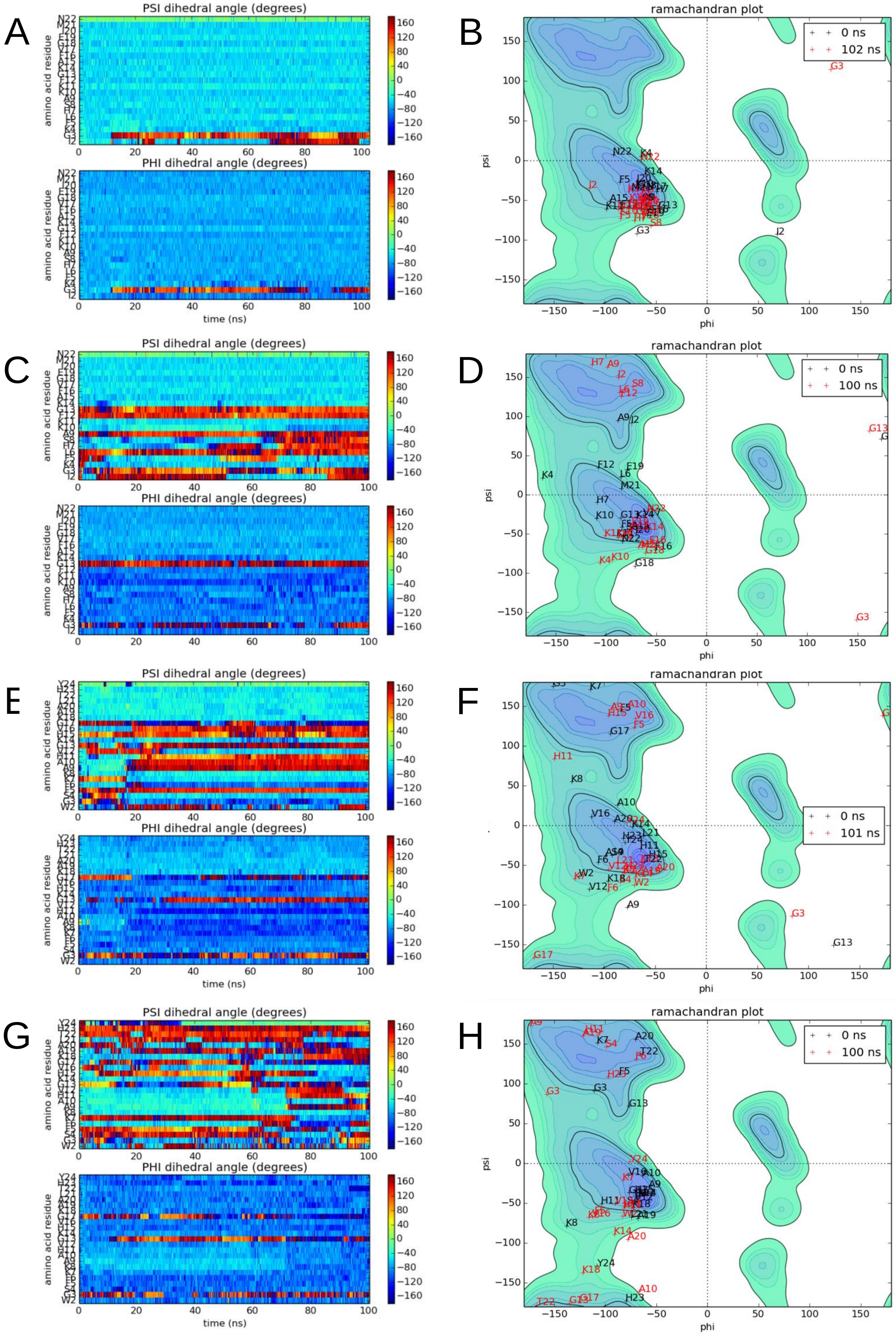

Sup. Fig. 2. Secondary structure analysis of the duplicated simulation of the binding of a single magainin 2 (A-D) or pleurocidin (E-H) molecule to a membrane consisting of 384 POPE and 128 POPG lipids. Ramachandran plots for snapshots obtained at the start and end of the 100 ns run are compared (B/D/F/H) while psi angles for individual peptide residues are also plotted against time (A/C/E/G).

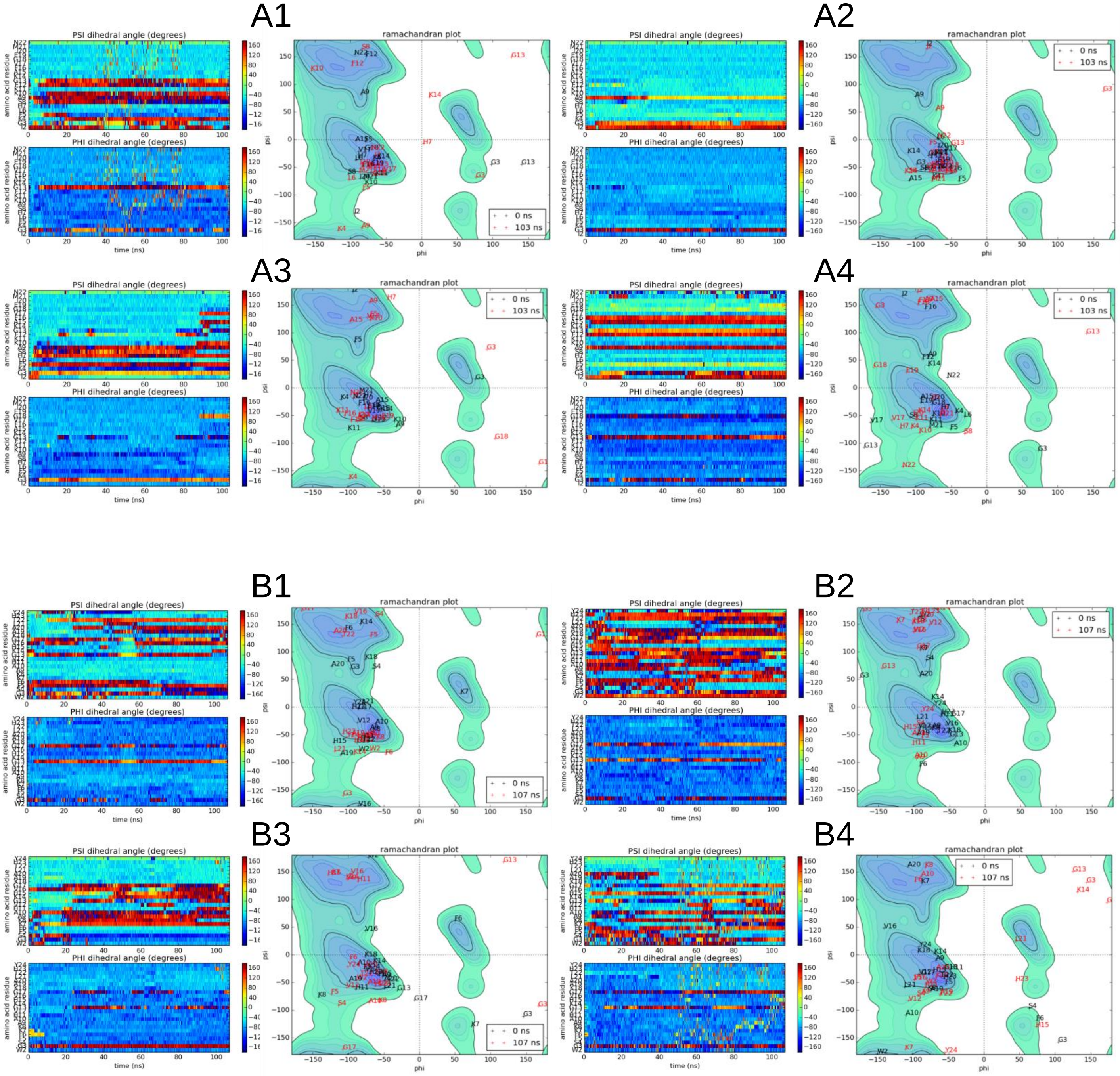

Sup. Fig. 3. Secondary structure analysis of the binding of four magainin 2 (A1-4) or four pleurocidin (B1-4) molecules to a membrane consisting of 384 POPE and 128 POPG lipids. Ramachandran plots for snapshots obtained at the start and end of 100 ns runs are compared (right hand panels) while psi angles for individual peptide residues are also plotted against time (left hand panels).

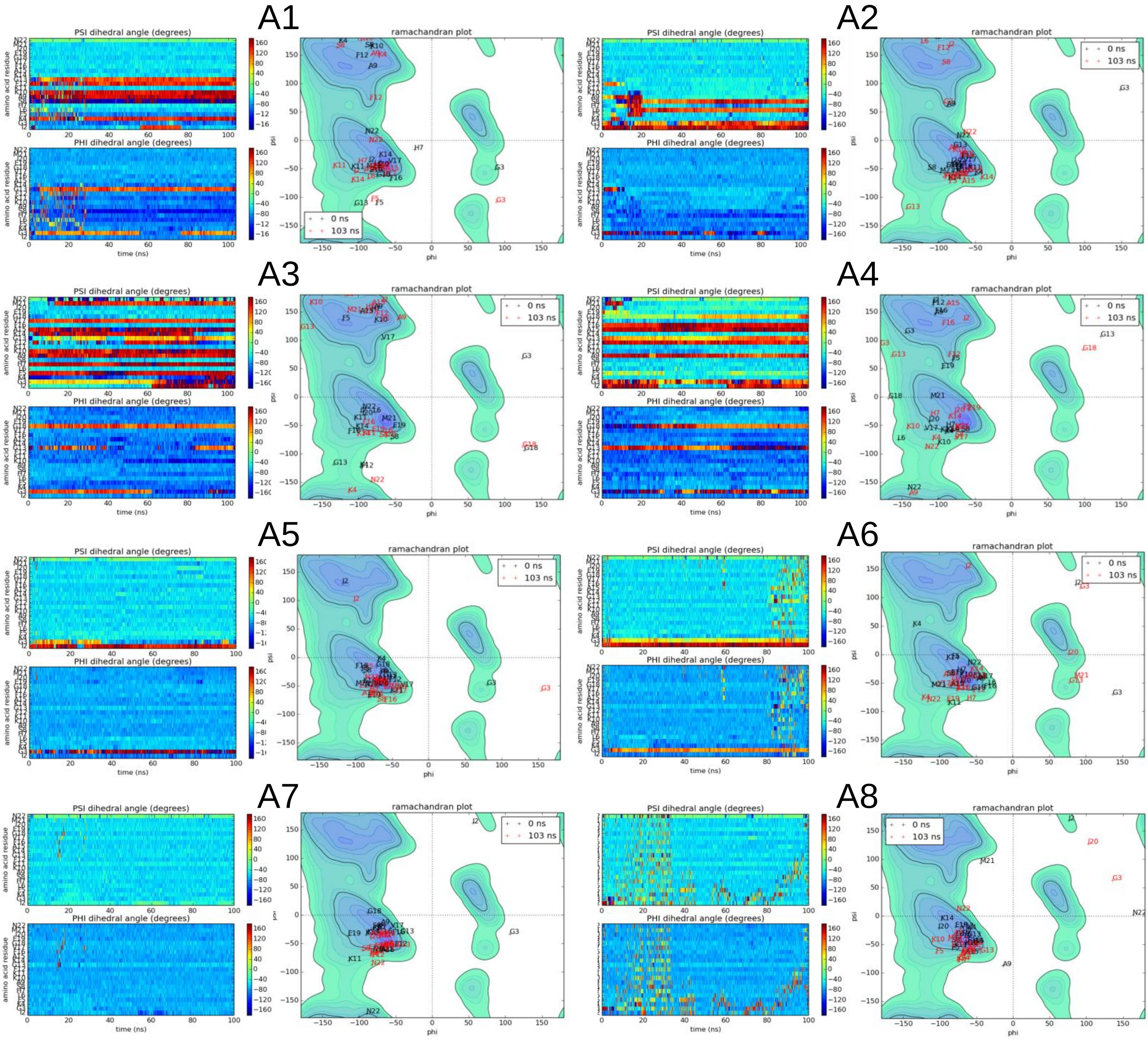

Sup. Fig. 4. Secondary structure analysis of the binding of eight magainin 2 (A1-8) molecules to a membrane consisting of 384 POPE and 128 POPG lipids. Ramachandran plots for snapshots obtained at the start and end of 100 ns runs are compared (right hand panels) while psi angles for individual peptide residues are also plotted against time (left hand panels).

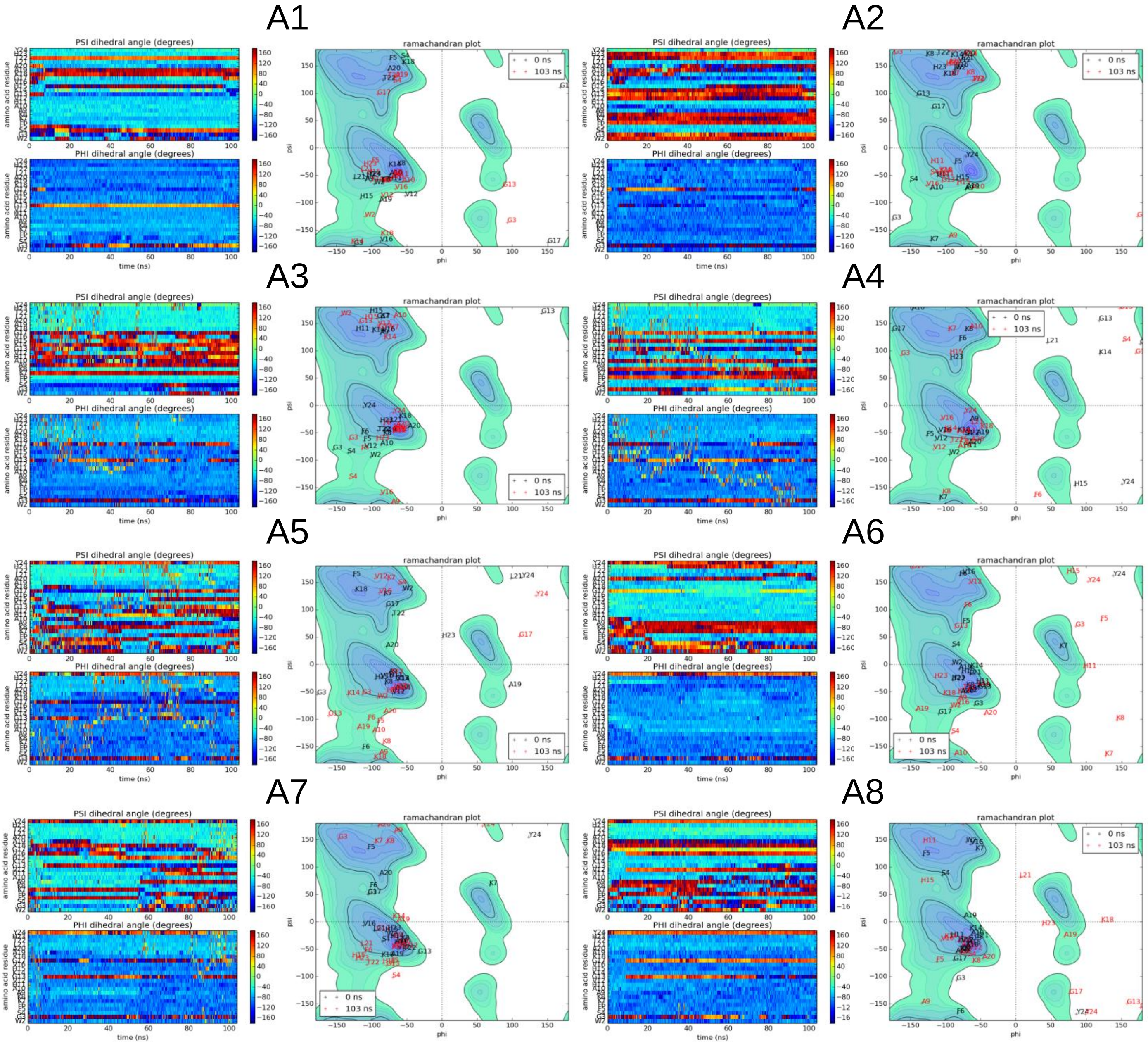

Sup. Fig. 5. Secondary structure analysis of the binding of eight pleurocidin (A1-8) molecules to a membrane consisting of 384 POPE and 128 POPG lipids. Ramachandran plots for snapshots obtained at the start and end of 100 ns runs are compared (right hand panels) while psi angles for individual peptide residues are also plotted against time (left hand panels).

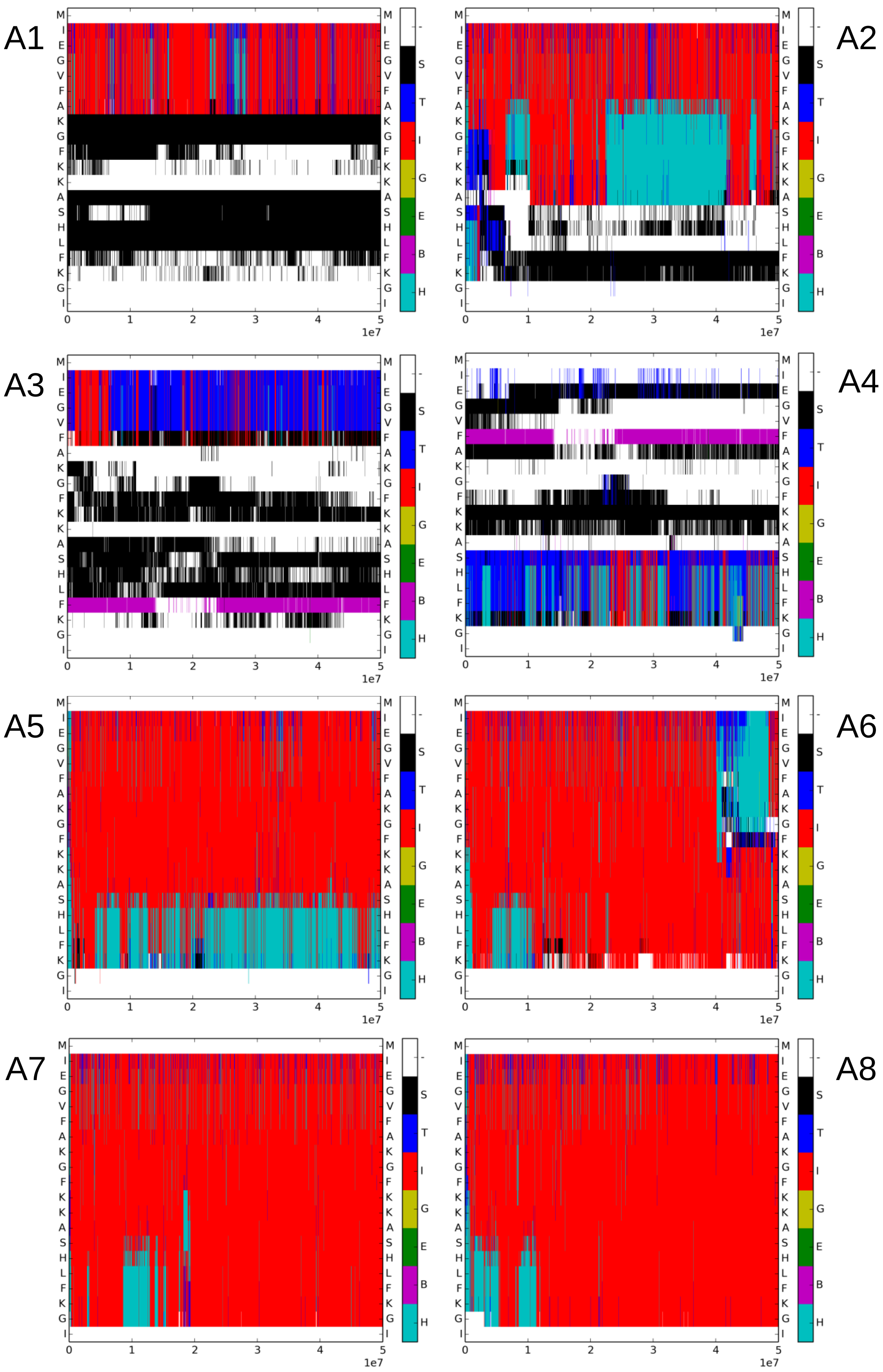

Sup. Fig. 6 DSSP secondary structure analysis of the binding of eight magainin 2 (A1-8) molecules to a membrane consisting of 384 POPE and 128 POPG lipids. Changes in assigned secondary structure are shown as a function of time step. The legend is according to the DSSP website [1]: H: helix, B: isolated beta bridge, E: extended strand in beta ladder, G: 3_10 helix, I: pi-helix, T: H-bonded turn, S: bend -: other.

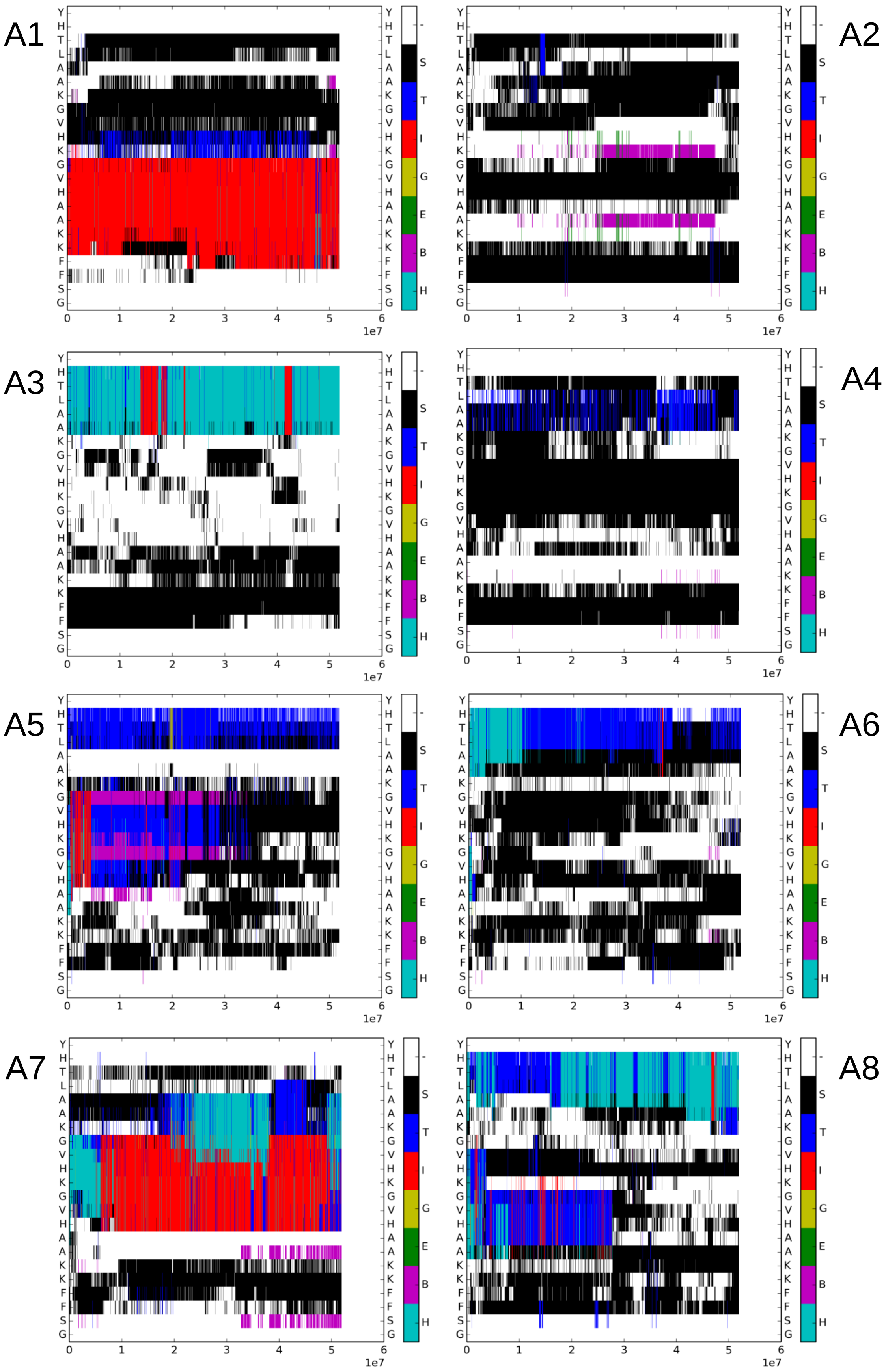

Sup. Fig. 7 DSSP secondary structure analysis of the binding of eight pleurocidin (A1-8) molecules to a membrane consisting of 384 POPE and 128 POPG lipids. Changes in assigned secondary structure are shown as a function of time step. The legend is according to the DSSP website [1]: H: helix, B: isolated beta bridge, E: extended strand in beta ladder, G: 3_10 helix, I: pi-helix, T: H-bonded turn, S: bend -: other.

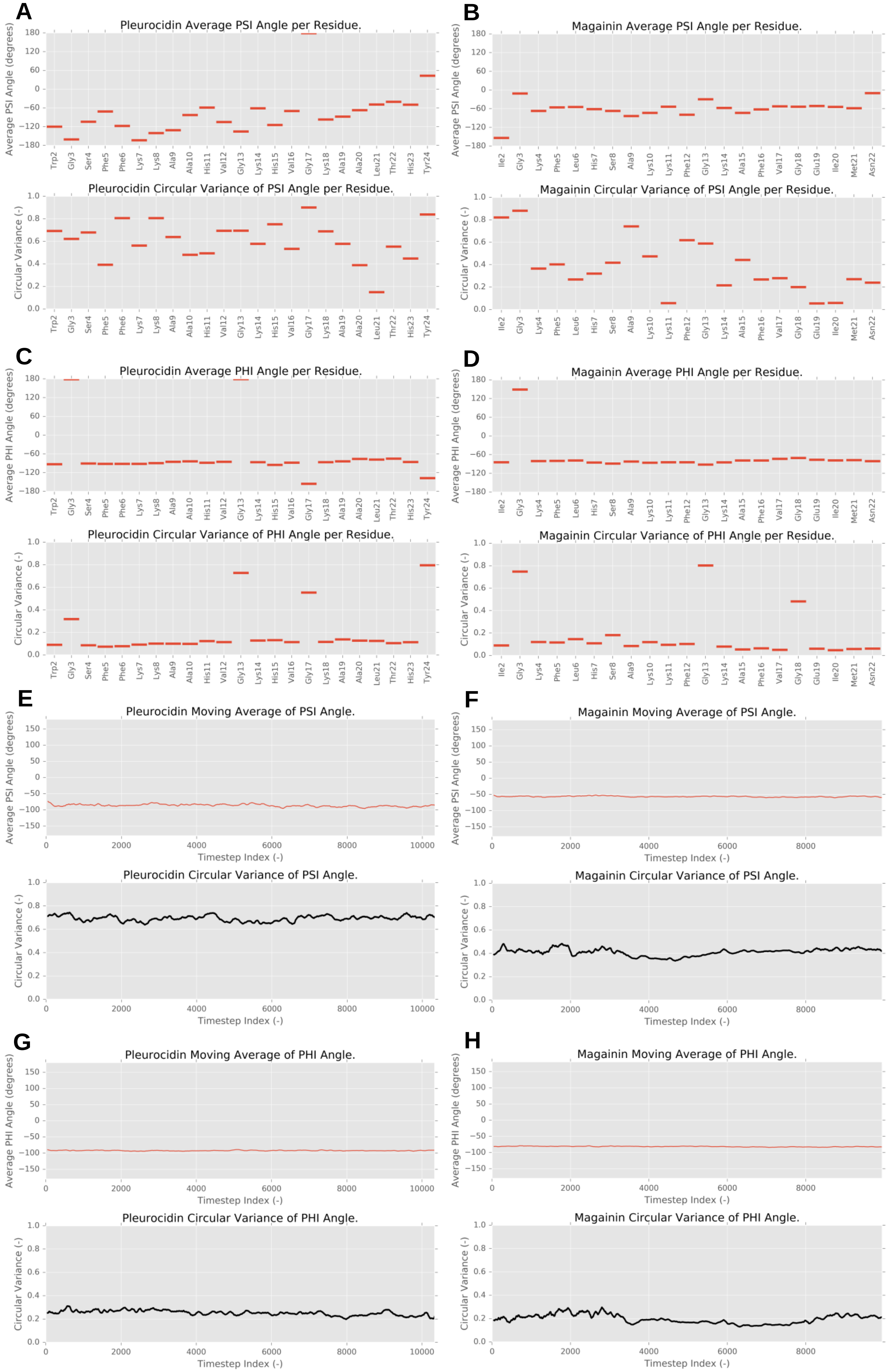

Supp. Fig. 8. Secondary structure analysis of the binding of eight pleurocidin (**A/C/E/G**) or magainin 2 (**B/D/F/H**) molecules to membranes consisting of 128 POPG and 384 POPE lipids. Psi (**A/B/E/F**) and phi (**C/D/G/H**) angles for individual peptide residues (**A-D**) or the peptide average (**E-H**) are averaged over the eight peptides and 100 ns of each simulation.

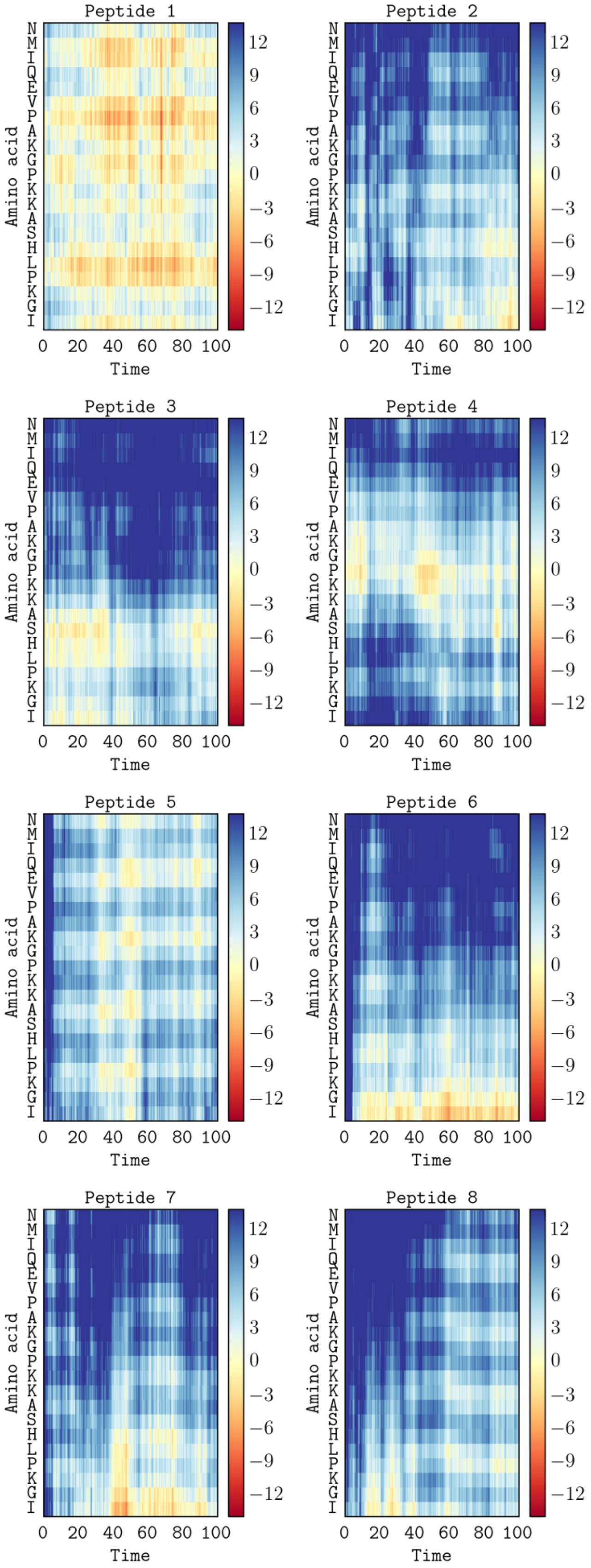

Supp. Fig. 9. The distance to the upper membrane leaflet phosphate plane of each amino acid is plotted over 100 ns for each of the eight magainin 2 peptides in the eight peptide/512 lipid simulation. Colours indicate distances (Å) for each residue above or below the plane of the lipid phosphates in the upper membrane leaflet.

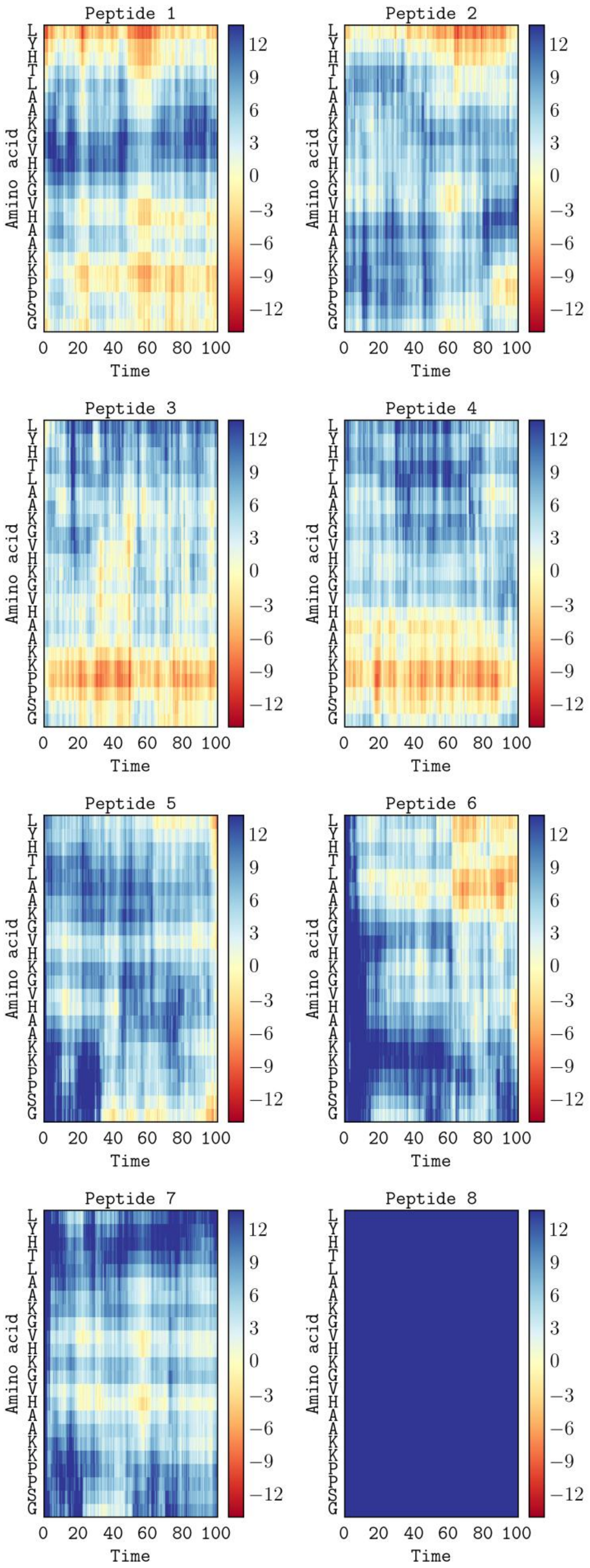

Supp. Fig. 10. The distance to the upper membrane leaflet phosphate plane of each amino acid is plotted over 100 ns for each of the eight pleurocidin peptides in the eight peptide/512 lipid simulation. Colours indicate distances (Å) for each residue above or below the plane of the lipid phosphates in the upper membrane leaflet. Peptide 8 did not bind to the membrane and migrated through the periodic boundary.

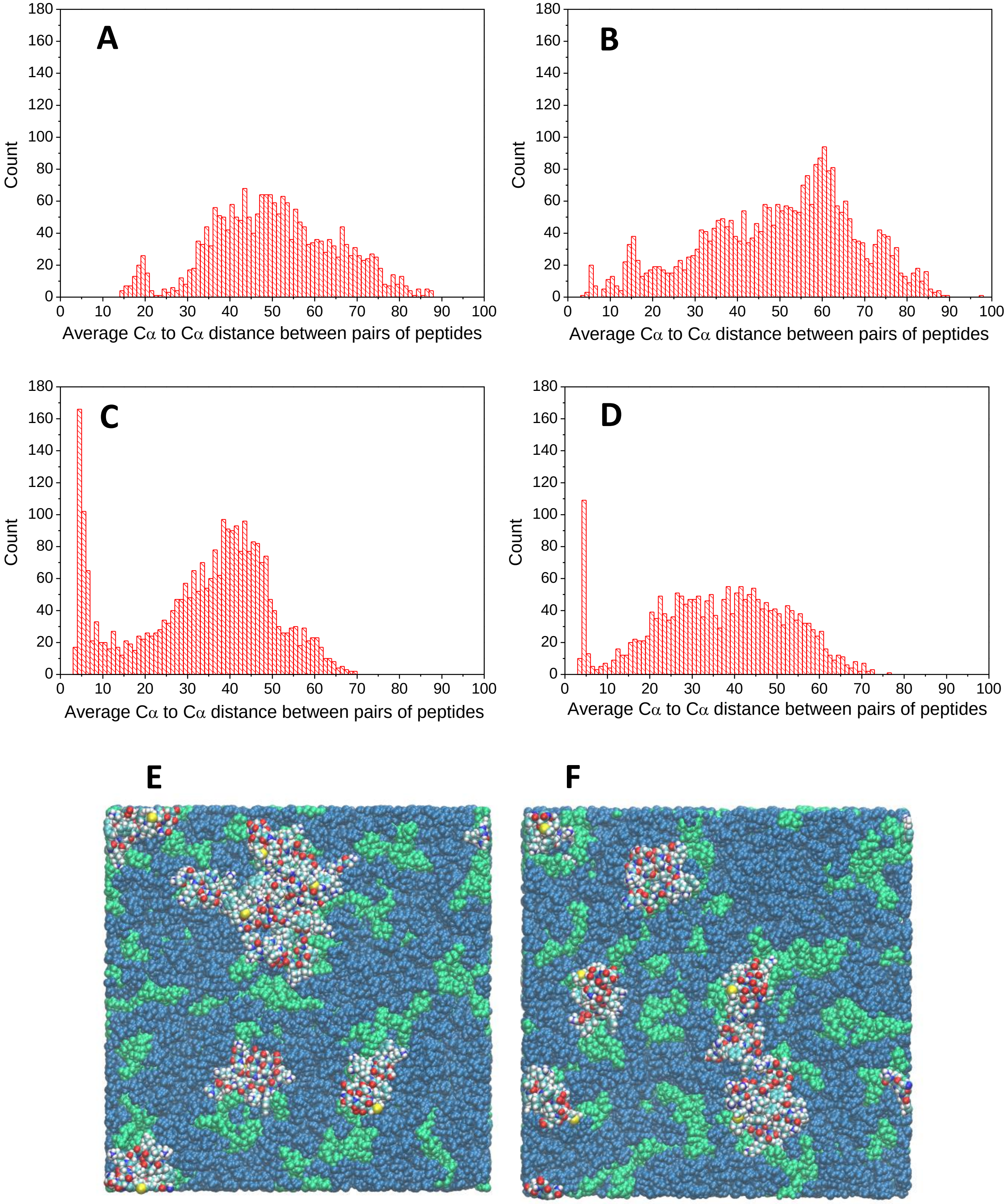

Sup. Fig. 11 The average distance frequency between Cα atoms in pairs of peptides is plotted for each ns of the 100 ns for the duplicate pleurocidin (**A**, **B**) and magainin 2 (**C**, **D**) in simulations with 8 peptides**.** The data for the pleurocidin (**A**) and duplicate magainin 2 simulation (**D**) does not include the one peptide in each simulation that broke the boundary conditions and migrated to the opposing leaflet.

Top view snapshots of duplicate simulations for eight magainin 2 peptides in POPE/POPG membranes at 100 ns, showing self-association of magainin 2 (**E**, **F**).

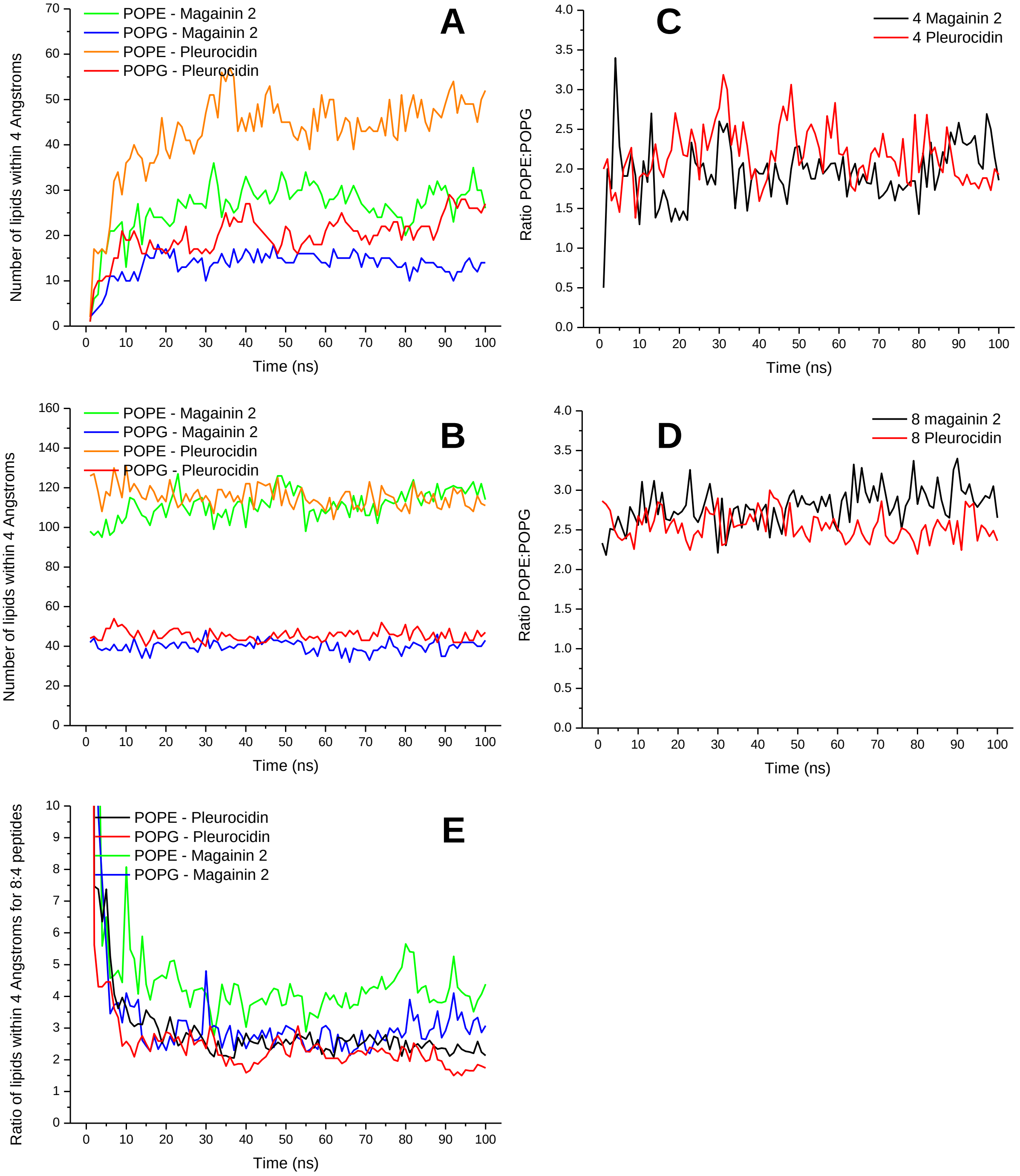

Sup. Fig. 12. **A)** 4 peptide simulations showing the number of lipid atoms of each type within 4 Å of peptide over 100 ns. **B)** 8 peptide simulations showing the number of lipid atoms within 4 Å of peptide over 100 ns. **C)** and **D)** show the ratio of PE to PG lipid atoms within 4Å for the 4 peptide and 8 peptide simulations respectively. **E)** Ratio of (B) to (A) revealing the concentration dependent and independent activity of magainin2 and pleurocidin respectively.

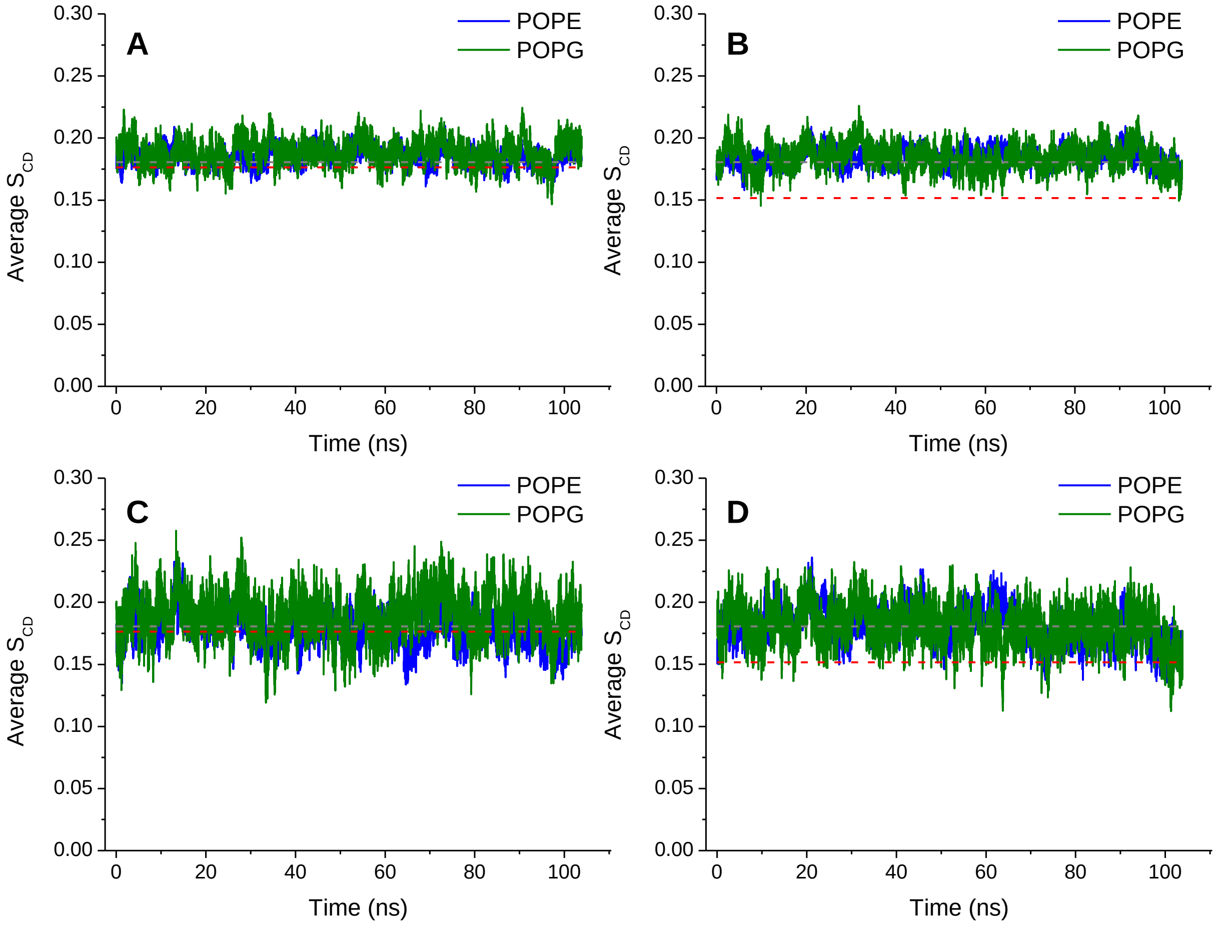

Supp. Fig. 13. Average order parameters of magainin 2 (**A/C**) and pleurocidin (**B/D**) eight peptide simulations for all lipids (**A/B**) or those lipids within 4 angstroms of any peptide (**C/D**). In each case the average deuterium order parameter (S_{CD}) for POPG-d31, determined experimentally by ^{2}H NMR, is shown for peptide free (grey line) and peptide challenged (red line) membranes.

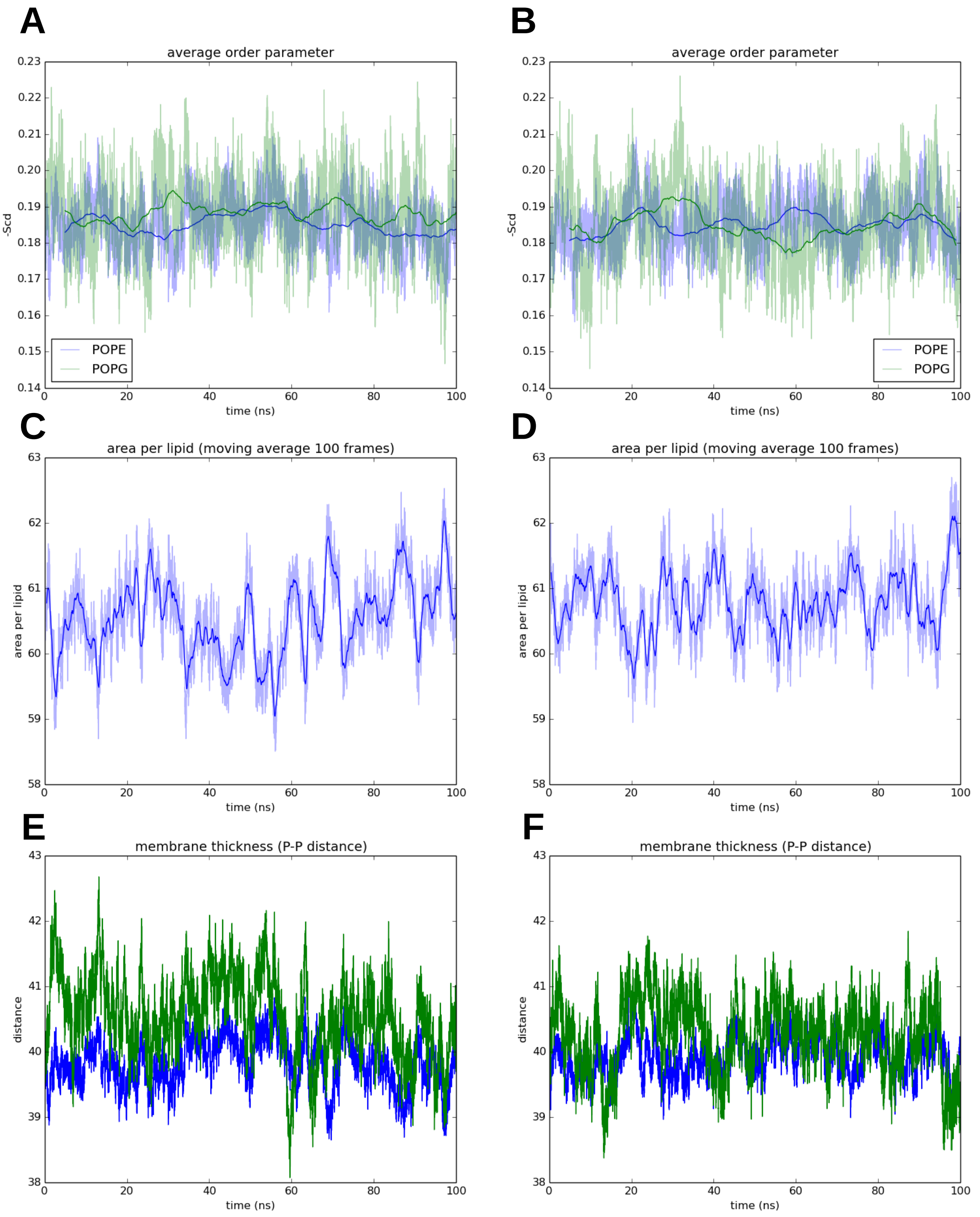

Sup. Fig. 14 Membrane properties of POPE/POPG membrane simulated with eight peptides of magainin 2 **(left)** and pleurocidin **(right)**. **(A,B)** The average order parameter over time with a smoothed average over 1000 frames for POPE (blue) and POPG (green). **(C,D)** The area per lipid as a moving average over 100 frames over time. **(E,F)** membrane thickness, measured as the distance between average phosphate position, over time for POPE (blue) and POPG (green).
